# Obesity modulates NK cell activity via LDL and DUSP1 signaling for populations with adverse social determinants

**DOI:** 10.1172/jci.insight.180606

**Published:** 2024-12-24

**Authors:** Yvonne Baumer, Komudi Singh, Abhinav Saurabh, Andrew S. Baez, Cristhian A. Gutierrez-Huerta, Long Chen, Muna Igboko, Briana S. Turner, Josette A. Yeboah, Robert N. Reger, Lola R. Ortiz-Whittingham, Sahil Joshi, Marcus R. Andrews, Elizabeth M. Aquino Peterson, Christopher K.E. Bleck, Laurel G. Mendelsohn, Valerie M. Mitchell, Billy S. Collins, Neelam R. Redekar, Skyler A. Kuhn, Christian A. Combs, Mehdi Pirooznia, Pradeep K. Dagur, David S.J. Allan, Daniella M. Schwartz, Richard W. Childs, Tiffany M. Powell-Wiley

**Affiliations:** 1 Social Determinants of Obesity and Cardiovascular Risk Laboratory,; 2 Bioinformatics and Computational Core Facility,; 3 Section of Transplantation Immunotherapy, Cellular and Molecular Therapeutics Branch, and; 4 Electron Microscopy Core Facility, National Heart, Lung and Blood Institute, NIH, Bethesda, Maryland, USA.; 5 Integrative Data Sciences Section, National Institute of Allergy and Infectious Diseases, NIH, Bethesda, Maryland, USA.; 6 Light Microscopy Core and; 7 Flow Cytometry Core, National Heart, Lung and Blood Institute, NIH, Bethesda, Maryland, USA.; 8 Division of Rheumatology and Clinical Immunology, University of Pittsburgh, Pittsburgh, Pennsylvania, USA.; 9 Intramural Research Program, National Institute on Minority Health and Health Disparities, NIH, Bethesda, Maryland, USA.

**Keywords:** Cardiology, Immunology, Lipoproteins, NK cells, Obesity

## Abstract

African American (AA) women are disproportionately affected by obesity and hyperlipidemia, particularly in the setting of adverse social determinants of health (aSDoH) that contribute to health disparities. Obesity, hyperlipidemia, and aSDoH appear to impair NK cells. As potential common underlying mechanisms are largely unknown, we sought to investigate common signaling pathways involved in NK cell dysfunction related to obesity and hyperlipidemia in AA women from underresourced neighborhoods. We determined in freshly isolated NK cells that obesity and measures of aSDoH were associated with a shift in NK cell subsets away from CD56^dim^/CD16^+^ cytotoxic NK cells. Using ex vivo data, we identified LDL as a marker related to NK cell function in an AA population from underresourced neighborhoods. Additionally, NK cells from AA women with obesity and LDL-treated NK cells displayed a loss in NK cell function. Comparative unbiased RNA-sequencing analysis revealed *DUSP1* as a common factor. Subsequently, chemical inhibition of Dusp1 and Dusp1 overexpression in NK cells highlighted its significance in NK cell function and lysosome biogenesis in a mTOR/TFEB-related fashion. Our data demonstrate a pathway by which obesity and hyperlipidemia in the setting of aSDoH may relate to NK cell dysfunction, making DUSP1 an important target for further investigation of health disparities.

## Abstract 2

Obesity and hyperlipidemia in the setting of adverse social determinants negatively impact NK cells via a pathway involving DUSP1/mTOR/TFEB, contributing to increased heart disease risk.

## Graphical abstract

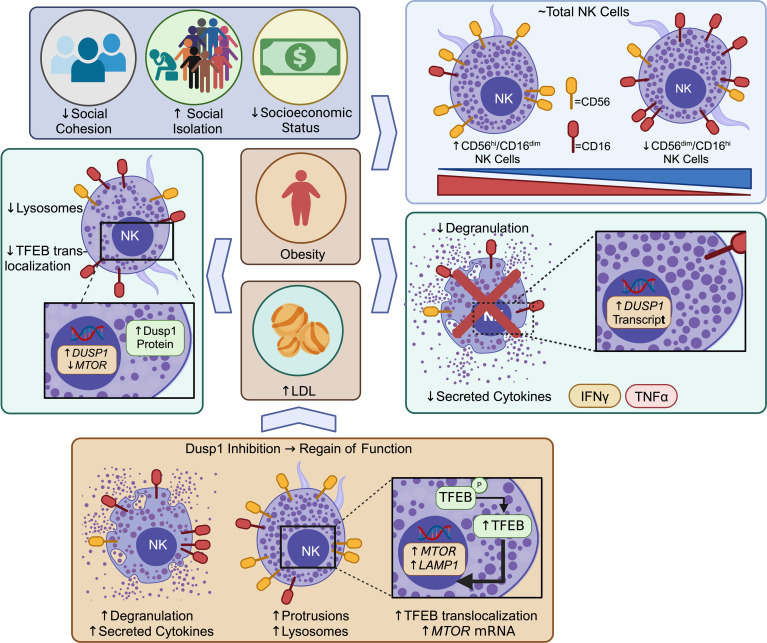

## Introduction

Obesity, defined by the World Health Organization as excessive or abnormal accumulation of adipose tissue, is associated with distinct health risks and has a complex pathogenesis involving biological and social factors at multiple levels (1). Social determinants of health (SDoH) play a crucial role in the development of obesity; these SDoH include neighborhood environment, socioeconomic status (SES), and psychosocial factors (2). Furthermore, adverse SDoH (aSDoH) can impede the treatment of individuals with obesity and magnify the racial and ethnic disparities seen in obesity and related comorbidities (3). After age adjustment, obesity is most prevalent in non-Hispanic Black populations, followed by Hispanic and non-Hispanic White populations, with non-Hispanic Black women at the highest risk for incident obesity (3).

Obesity increases the risk of cardiovascular disease (CVD), hyperlipidemia, type 2 diabetes, and some cancers (4, 5), reducing life expectancy by 5–20 years (4). Many of these outcomes have been linked to excess obesity-related inflammation. Increased visceral adiposity promotes the release of proinflammatory mediators, including TNF-α and IL-6 (6). These adipokines, cytokines, and chemokines modulate hematopoiesis (7) and immunity (5, 8) to promote adverse obesity-related outcomes.

Obesity can affect the phenotype and function of almost any immune cell subset (9). Many prior studies have focused on monocytes (10) and neutrophils (11) owing to their critical role in atherogenesis (12, 13). Yet NK cells — a subset of innate-like lymphocytes with cytolytic function — may also regulate atherogenesis (14). NK cells are also important for early host-pathogen responses, cancer surveillance, immunomodulation, and cellular cytotoxicity (15). NK cell functional subsets are categorized by CD56 expression, with highly proliferative CD56^bright^CD16^–^ NK cells acting as immunomodulators, while CD56^dim^CD16^+^ NK cells are more cytotoxic (16).

Class III obesity (BMI ≥40 kg/m^2^) has been associated with metabolic reprogramming of NK cells, reduced numbers of cytotoxic CD3^–^/CD56^+^ NK cells, and reduced NK cell function (17). In children with obesity and insulin resistance, reduced NK cell numbers were also accompanied by defective tumor lysis (18). Adipose tissue–resident NK cells can express lower levels of activating NKp30 and NKp44 receptors in individuals with obesity (19). Together, these findings suggest that obesity modulates NK cell distribution and function (17, 20, 21). Yet the specific pathways modulating NK cell alterations in individuals with obesity are incompletely characterized. This is especially true in marginalized populations with obesity, who disproportionately experience aSDoH and are traditionally underrepresented in research studies.

Herein, we examined the effects of obesity on the phenotype and function of circulating NK cells. To specifically investigate the effects of obesity in the setting of aSDoH, we studied NK cells in a cohort of African American (AA) women from resource-limited neighborhoods, comparing age-matched individuals with obesity versus those without obesity. As we have previously demonstrated that LDL regulates immune cell function (22), we explored pathways modulated by both LDL and obesity for further analysis. Furthermore, prior studies suggest that individuals experiencing aSDoH are less likely to receive lipid-lowering therapies (23, 24), highlighting the potential intersectionality and interconnectedness of obesity and hyperlipidemia for populations most exposed to aSDoH. We hypothesized that pathways related to obesity- and hyperlipidemia-mediated changes in NK cell phenotype influence NK cell function, promoting CVD, cancer, and other obesity-related comorbidities.

## Results

### The frequency of NK cell subsets in blood of AA female individuals as related to obesity in the setting of aSDoH.

**Figure 1:**
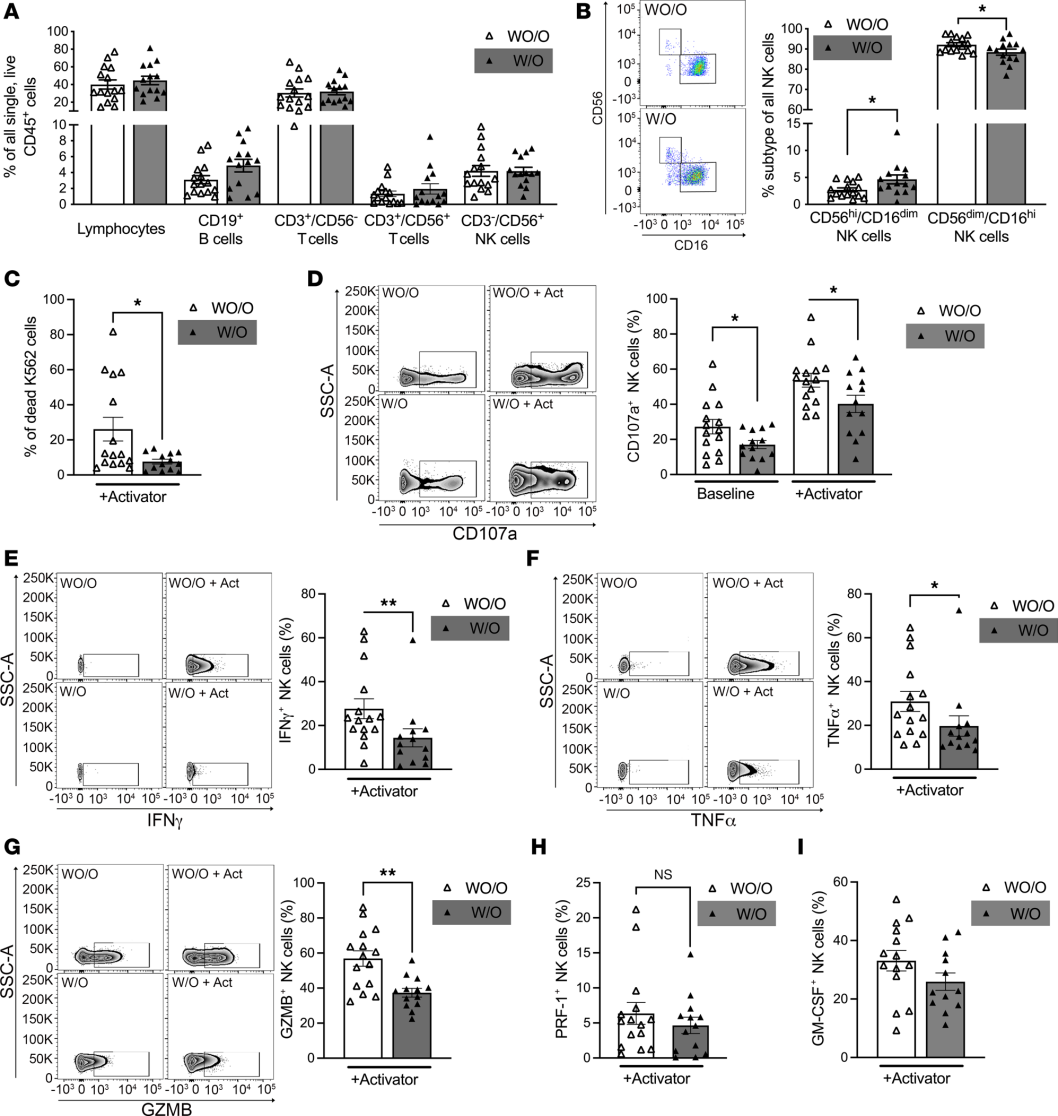
Obesity associated with differences in NK cell subset profiles and NK cell dysfunction. Age-matched AA female individuals from resource-limited neighborhoods who did (W/O; BMI ≥ 30 *n* = 14) or did not present with obesity (WO/O; BMI < 30, *n* = 15), respectively, were study participants. (**A** and **B**) Whole blood was examined by flow cytometry to analyze the lymphocytes, namely, CD19^+^ B, CD3^+^/CD56^–^ T, CD3^+^/CD56^+^ T, and CD3^–^/CD56^+^ NK cell populations. (**B**) Deeper phenotyping of CD56^+^ NK cells by CD16 expression allows a general identification of CD56^hi^/CD16^dim/–^ and CD56^dim^/CD16^hi^ NK cell phenotypes. (Depending on normality test, either 2-tailed unpaired *t* test or Mann-Whitney test comparing 2 patient groups for each immune cell population was performed.) (**C**–**I**) NK cells were isolated from AA female individuals who did (*n* = 13) or did not present with obesity (*n* = 15) and were subjected to the degranulation assay under baseline and activating conditions. For activating conditions (+activator), NK cell Cell Activator Cocktail was added right before the experiment to determine the maximum response of the tested conditions. By using flow cytometry, the proportion of dead K562 cells (**C**), as a measure of NK cell killing ability under activating conditions; the expression of CD107a (**D**), as a measure of NK cell degranulation; and the intracellular expression of IFN-γ (**E**), TNF-α (**F**), granzyme B (**G**), perforin (**H**), and GM-CSF (**I**) were detected. Statistical significance was determined by using (**C**, **E**, **F**, and **H**) Mann-Whitney test and (**D**, **G**, and **I**) unpaired (2-tailed) *t* test. Significance was established at *P* < 0.05, comparing individual groups with the data-appropriate test; asterisks indicate significance between groups. Flow data are accompanied by representative dot or volcano blots. *P < 0.05; **P < 0.01.

Between 29 age-matched AA female individuals from resource-limited neighborhoods with obesity (W/O, *n* = 14, BMI ≥30 kg/m^2^) or without overweight/obesity (WO/O, *n* = 15, BMI <30 kg/m^2^) (Supplemental Table 1), there was no significant difference in the frequency of lymphocytes overall (44.7% ± 4.8% vs. 40.1% ± 5.1%), CD19^+^ B cells (4.9% ± 0.8% vs. 3.1% ± 0.5%), CD3^+^/CD56^–^ T cells (31.9% ± 3.5% vs. 30.4 ± 4.6%), CD3^+^ expressing CD56 T cells (1.9% ± 0.7% vs. 1.3% ± 0.4%), and CD3^–^/CD56^+^ NK cells (4.2% ± 0.5% vs. 4.2% ± 0.7%) within circulating CD45^+^ mononuclear cells (Figure 1A and Supplemental Figure 1). We observed a significantly higher proportion of CD56^bright^/CD16^dim^ NK cells (4.6% ± 0.8% versus 2.7% ± 0.4%; *P* = 0.029) and significantly lower proportion of CD56^dim^/CD16^+^ NK cells (88.5% ± 1.5% versus 92.1% ± 1.0%; *P* = 0.048) within the overall CD3^–^/CD56^+^ NK cell population in W/O AA female individuals compared with age-matched WO/O AA female individuals (Figure 1B).

Within this cohort of 29 AA female individuals from resource-limited neighborhoods, data evaluating psychosocial factors (i.e., social isolation, depressive symptoms) and SES as aSDoH were available for 17 study participants (Supplemental Table 1). As an exploratory analysis, we examined associations between aSDoH and total, CD56^bright^/CD16^dim/–^, and CD56^dim^/CD16^+^ NK cells for these 17 study participants (Table 1). These 17 study participants were at intermediate risk for CVD and, on average, had class I obesity (Supplemental Table 1). Higher social isolation was associated with lower CD56^dim^/CD16^+^ NK cell proportions (β = –0.69, *P* = 0.02 in BMI/atherosclerotic cardiovascular disease 10-year risk–adjusted [BMI/ASCVD-adjusted] models). Higher depressive symptoms trended toward a significant association with lower total NK cells (β = –0.47, *P* = 0.09 in BMI/ASCVD-adjusted models). This association was likely driven by the CD56^dim^/CD16^+^ NK cell proportions (β = –0.54, *P* = 0.05 in BMI/ASCVD-adjusted models) (Table 1). Higher individual-level SES, as measured by household income, was also associated with higher CD56^dim^/CD16^+^ NK cell proportions (β = 0.65, *P* = 0.02 in BMI/ASCVD-adjusted models) (Table 1). After multicomparison adjustment, the associations between aSDoH and CD56^dim^/CD16^+^ NK cell proportions remained significant.

### Obesity is associated with reduced NK cell degranulation and cytokine secretion.

Because obesity was associated with an increase in regulatory CD56^bright^/CD16^dim/–^ cells and a decrease in the cytotoxic subset CD56^dim^/CD16^+^ NK cells, and changes in NK cell proportions do not necessarily translate into NK cell function changes, we examined whether cytotoxic function might also be affected by obesity status. Therefore, we compared the degranulation and cytotoxic capacity of NK cells from age-matched AA female individuals W/O and WO/O. NK cells isolated from age-matched AA female individuals W/O displayed a 71% decreased ability to kill target K562 cells compared with NK cells from AA female individuals WO/O (Figure 1C). Additionally, the surface expression of CD107a, a degranulation marker, was 25% lower in NK cells of AA female individuals W/O compared with females WO/O (Figure 1D). Under NK cell–activating conditions and after contact with susceptible K562 target cells, we detected significantly lower levels of intracellular granzyme B (34% decrease), IFN-γ (52% decrease), and TNF-α (64% decrease) in NK cells from age-matched AA female individuals W/O compared with AA female individuals WO/O (Figure 1, E–G). We did not see significant changes in intracellular perforin or GM-CSF production (Figure 1, H and I).

### LDL impairs NK cell function in vitro and in the setting of obesity in women exposed to aSDoH.

**Figure 2:**
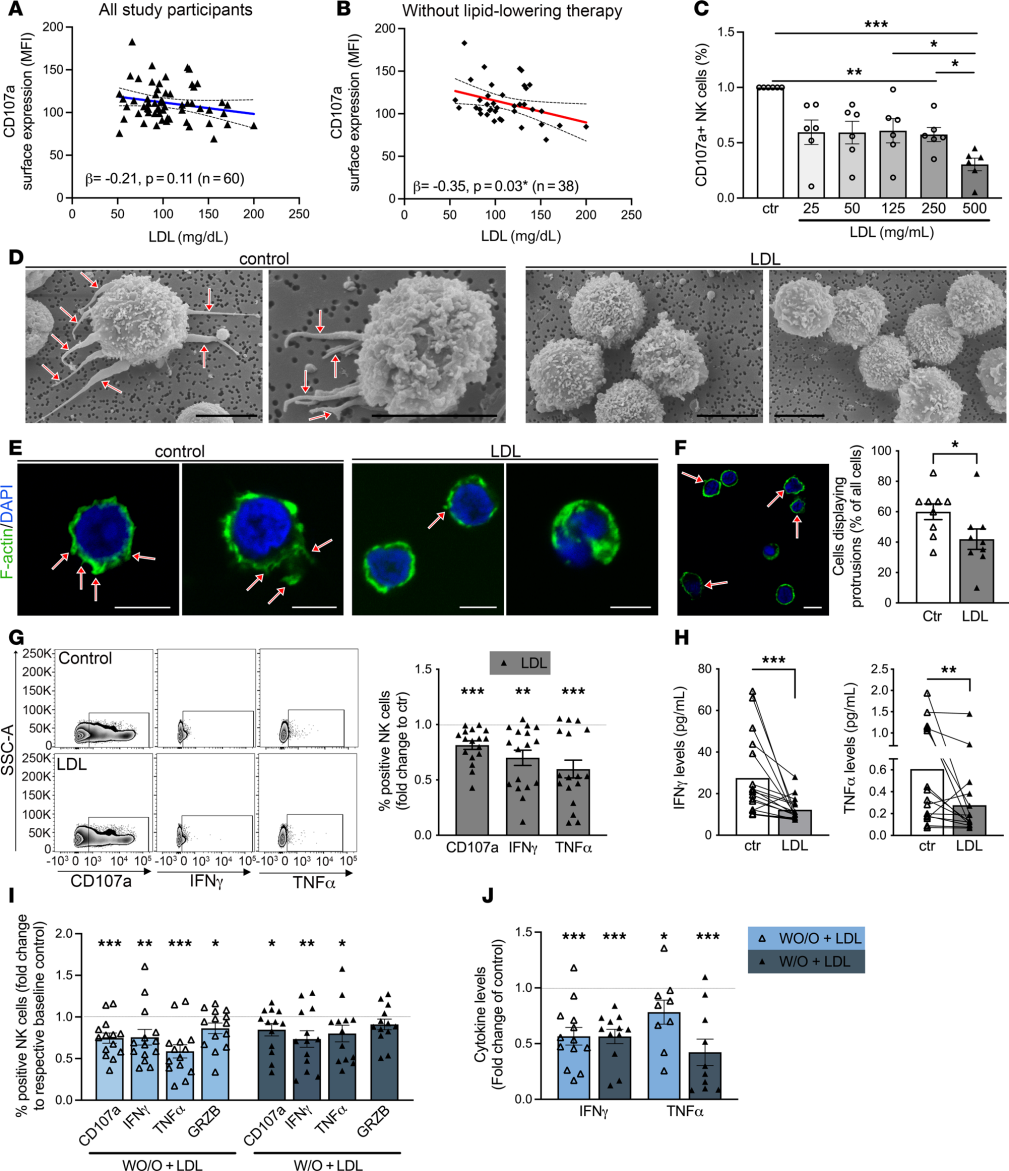
LDL is associated with NK cell dysfunction in the setting of obesity and adverse social determinants and in vitro experiments. (**A** and **B**) NK cells were isolated from a healthy blood bank donor, treated overnight with study participants’ sera (*n* = 60), and subsequently subjected to the degranulation assay toward K562 cells. Surface CD107a expression levels were utilized in multivariable regression analysis against LDL levels of the study participants. (**A**) Data from all 60 study participants. (**B**) Data from participants without lipid-lowering therapy (subgroup *n* = 38). (**C**) Freshly isolated primary NK cells from blood bank donors were incubated with increasing doses of LDL, and the effect on degranulation (CD107a) after exposure to K562 cells was measured using flow cytometry. (**D**–**J**) NK cells from healthy blood bank donors were treated overnight with LDL (50 mg/dl) or vehicle control. (**D**) Utilizing scanning electron microscopy, the presence of protrusions (arrows) was examined as a sign of NK cell activity (*n* = 3). (**E** and **F**) Labeling the actin cytoskeleton of treated NK cells using FITC-Phalloidin with subsequent confocal microscopy imaging allowed for quantification of cells presenting protrusions (*n* = 9, unpaired 2-tailed *t* test). (**G** and **H**) Degranulation assay was performed after overnight treatment (**G**,*n* = 17, Mann-Whitney test). Supernatants were subjected to ELISA-based measurement of secreted cytokines (**H**) (*n* = 17, Wilcoxon test for each cytokine set). (**I** and **J**) NK cells were isolated from age-matched AA female participants with (W/O) or without (WO/O) obesity (BMI ≥ 30, *n* = 13 and BMI < 30, *n* = 14), treated with LDL overnight and subsequently subjected to the degranulation assay (**I**), and the levels of secreted cytokines were determined by ELISA (IFN-γ, WO/O, *n* = 13 and W/O, *n* = 12; TNF-α, WO/O, *n* = 9 and W/O, *n* = 10) (**J**). Significance was established at *P* < 0.05, comparing individual groups with an unpaired *t* test (**I**, **F**, and **G**) or Mann-Whitney test (**I**, **H**, and **J**) dependent on dataset normality test; all tests were 2 tailed; asterisks indicate significance between groups. *P < 0.05; **P < 0.01; ***P < 0.001. Scale bar: 5 μm.

To understand how lipids might impact NK cell function in the setting of obesity and aSDoH, we utilized an ex vivo approach. We examined the influence of serum lipids from a cohort of AA individuals from underresourced neighborhoods (mean BMI = 33 ± 7.9 kg/m^2^, *n* = 60) on healthy donor NK cells (cohort 2, Supplemental Table 2). After 24 hours of treatment, we performed the degranulation assay toward K562 cells. Subsequently, these results were subjected to multivariable regression analysis with the levels of various serum lipid components in these samples (Table 2, Supplemental Table 3, and Figure 2, A and B). No significant associations were found between levels of HDL or ApoA1 and NK cell degranulation, as measured by CD107a expression. Higher LDL and ApoB levels were associated with lower CD107a expression among study participants on no lipid-lowering therapy (Table 2, and Figure 2, A and B). We also found that higher serum total cholesterol, LDL, and ApoB were associated with lower intracellular IFN-γ expression independent of ASCVD risk, BMI, and SES among participants on no lipid-lowering therapy.

Given that serum LDL was associated with NK cell CD107a and IFN-γ expression in the ex vivo experiments, we investigated the presence of a dose-dependent effect of LDL on NK cell degranulation in in vitro experiments. In prior studies, subphysiological levels of LDL were typically used to treat immune cells, including NK cells (25–27). More recently, our research has focused on physiological LDL concentrations in in vitro studies (22, 28–30). By testing various concentrations in freshly isolated NK cells from blood bank donors, we found that concentrations between 25 and 500 mg/mL (2.5–50 mg/dL) led to reduced degranulation, as measured by CD107a presence on NK cell surface after contact with K562 cells. We observed an additional and significant drop in NK cell degranulation at an LDL concentration of 50 mg/dL, which is close to the upper limit of plasma LDL concentration goals for very high CVD risk populations (≤ 55 mg/dL), based on the 2023 American College of Cardiology guidelines (31) (Figure 2C).

Therefore, we examined the potential effect of LDL (500 mg/mL = 50 mg/dL) on primary freshly isolated NK cells from blood bank donors and subsequently analyzed phenotypic and functional changes (Figure 2, D–H). First, we utilized scanning electron microscopy to examine NK cell morphology and protrusion presence, as these are crucial for the cytotoxic capabilities of NK cells (32). We found that LDL-treated NK cells displayed fewer protrusions compared with vehicle-treated NK cells (Figure 2D). These findings were confirmed utilizing *Z*-stack confocal microscopy of F-actin–labeled NK cells (Figure 2, E and F). Next, we measured NK cell degranulation and cytokine production following LDL exposure (Figure 2, G and H). LDL exposure decreased CD107a surface expression (*P* < 0.0001) compared with vehicle treatment. Intracellular NK cell IFN-γ and TNF-α were significantly decreased by LDL exposure (*P* = 0.0001 and *P* < 0.0001, respectively). Secretion of IFN-γ and TNF-α were significantly decreased by LDL exposure (*P* = 0.0008 and *P* = 0.005, respectively; Figure 2H).

To determine if these effects of LDL on healthy volunteer NK cells were also occurring in NK cells isolated from our age-matched study participants of AA female individuals W/O or WO/O (cohort 1), we exposed study participants’ NK cells with LDL and performed the degranulation assay (Figure 2, I and J). Both groups of NK cells displayed decreased surface CD107a expression and decreased intracellular expression of IFN-γ and TNF-α (Figure 2I). LDL also significantly decreased levels of secreted IFN-γ and TNF-α in the supernatant of W/O and WO/O NK cells (Figure 2J).

**Figure 3:**
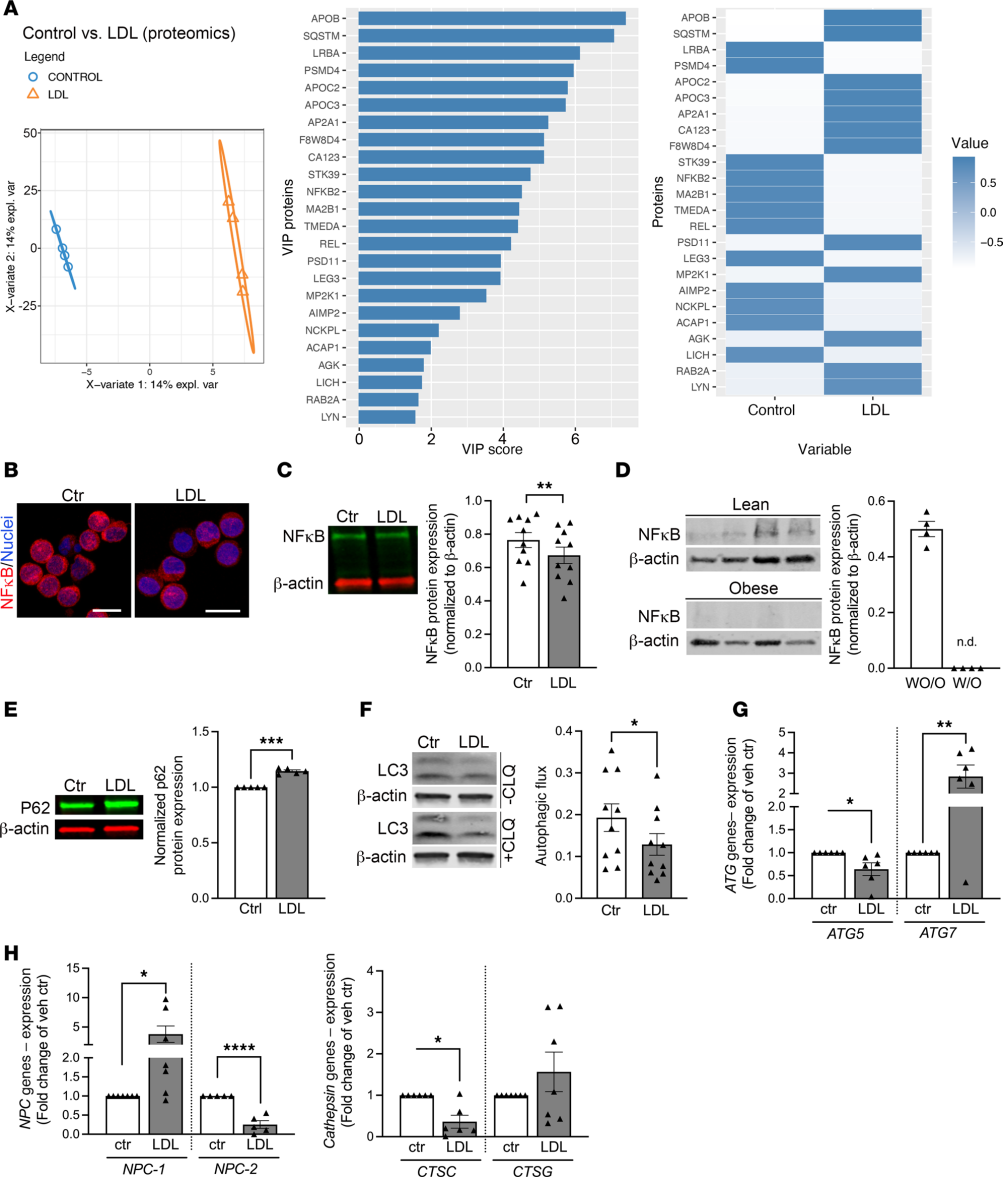
LDL treatment of NK cells affects various signaling pathways. NK cells were freshly isolated from blood bank donor buffy coats and subjected to vehicle control or LDL treatment overnight. (**A**) Proteomics analysis of vehicle control- and LDL-treated NK cells was performed. Partial least square discriminant analysis (PLSDA) plot of the proteomics results. Right: Variables important in predicting the control- and LDL-exposed NK cells. The variables of importance in prediction (VIP) score of the indicated discriminant proteins on the *y* axis and their contribution to control- and LDL-treated NK cells on the *x* axis are presented (*n* = 4 sets). (**B**) Freshly isolated primary NK cells were treated as indicated and subjected to immunofluorescence analysis of NF-κB (*n* = 3, red = NF-κB, blue = nucleus; scale bar: 10 μm). (**C**) Western blot analysis was performed. NF-κB expression was normalized to β-actin and summarized in the graph (*n* = 10, paired 2-tailed *t* test). (**D**) NK cells from study participants with or without obesity were subjected to Western blot analysis for NF-κB and β-actin. For individuals without obesity (WO/O), the expression of NF-κB was normalized to β-actin and displayed in the graph. No quantifiable NF-κB band was obtained for individuals with obesity (W/O) (*n* = 4 in each group). (**E**) Sqstm1/p62 expression was investigated in the vehicle- and LDL-treated NK cells with β-actin as the housekeeping protein. Results are displayed as fold change to control (*n* = 5, unpaired 2-tailed *t* test). (**F**) Autophagy flux was investigated by Western blotting. Data are shown as the flux for each treatment (*n* = 10, paired 2-tailed *t* test). (**G** and **H**) RT-qPCR analysis of genes important for autophagy (**G**) or lysosomal function (**H**). Data are shown as fold change to control (*n* = 6–7, unpaired 2-tailed *t* test for each pair). Significance was established at *P* < 0.05, comparing individual groups, and is indicated by asterisks. ATG, autophagy protein; CLQ, chloroquine; CTS, cathepsin; NPC, Nieman-Pick disease. *P < 0.05; **P < 0.01; ***P < 0.001.

We used proteomics analyses to characterize the mechanisms involved in LDL-induced NK cell function loss. Proteomic analysis (LC-MS/MS) of LDL-exposed NK cells identified 79 proteins (45 differentially induced and 34 repressed) that collectively differentiated LDL-exposed NK cells from the background, including NFKB2, which was significantly decreased (Figure 3A). We verified that NF-κB protein expression was significantly reduced in primary freshly isolated NK cells after LDL exposure using immunofluorescence analysis and Western blotting (Figure 3, B and C). When examining NK cells isolated from AA female individuals W/O and WO/O with Western blotting, we found decreased expression of NF-κB in NK cells isolated from AA women W/O (Figure 3D).

Proteomics analyses revealed additional variables of importance in prediction in LDL-exposed cells, including the autophagy protein p62 (SQSTM1), the lysosomal protein LICH (also known as lysosomal acid lipase [LAL]), and proteasomal complex proteins PSMD4 and PSD11 (Figure 3A). Western blotting analysis confirmed that the increased p62 expression in proteomics (Figure 3E) was accompanied by decreased autophagic flux in NK cells exposed to LDL (Figure 3F), suggesting a late-stage inhibition of the autophagy process in LDL-treated NK cells. Subsequently, we checked for LDL-associated changes in the expression of key genes known to regulate autophagy and lysosome function using RT-qPCR primary freshly isolated NK cells (Figure 3, G and H), establishing a phenotype displaying a dysregulation of lysosome function (*NPC1/2*, cathepsin C) with disturbed autophagy (*ATG5* vs. *ATG7*).

### Identification of DUSP1 involvement in NK cell dysfunction related to obesity and LDL.

**Figure 4:**
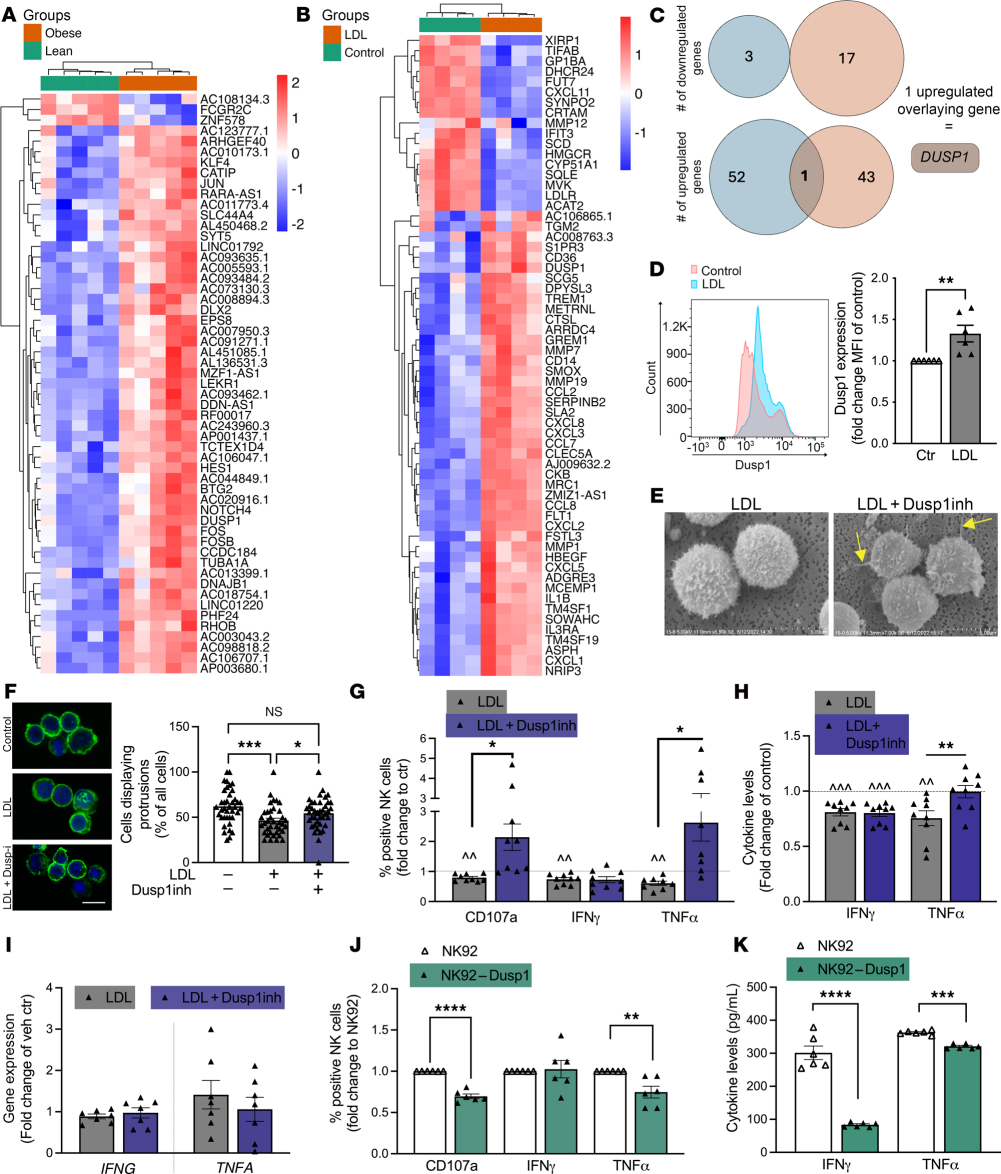
Comparative analysis of 2 RNA-sequencing sets reveals *DUSP1* as an overlapping significantly upregulated gene and important regulator of NK cell function. (**A**–**C**) Freshly isolated primary NK cells from age-matched AA women with (W/O) or without (WO/O) obesity (dataset 1, *n* = 5 each group) or healthy donors treated with vehicle or LDL overnight (dataset 2, *n* = 4) were subjected to RNA-sequencing analysis. (**A** and **B**) Heatmap of differentially expressed genes, significant at an adjusted *P* ≤ 0.1 and fold change (FC) ≥ 1.5 in (**A**) W/O vs. WO/O and (**B**) LDL vs. control comparisons. (**C**) Venn diagram showing the overlap of differentially expressed genes from 2 comparisons identified a single gene (*DUSP1*) being commonly upregulated. (**D**) Flow cytometry analysis of Dusp1 protein expression in NK cells with indicated treatments (*n* = 6, unpaired 2-tailed *t* test). (**E**–**I**) Freshly isolated primary NK cells were treated with LDL with or without Dusp1 inhibitor. (**E**) Scanning electron microscopy (SEM) imaging of treated NK cells (*n* = 3). Arrows point to protrusions of NK cells. Original magnification, ×8,500 (left); ×7,000 (right) (scale bar: 5 μm). (**F**) Labeling F-actin (FITC-Phalloidin) allowed for the quantification of cells presenting protrusions (*n* = 6 independent experiments with at least 5 visual fields analyzed per condition; Kruskal-Wallis test with Dunn’s correction) (scale bar: 5 μm). (**G**) Flow cytometry–based detection of degranulation (CD107a) and intracellular cytokine presence after exposure to K562 cells (*n* = 9, CD107a/IFN-γ; *n* = 8, TNF-α; repeated-measures 1-way ANOVA with Tukey’s correction). (**H**) Cytokine release was analyzed from supernatants. (**I**) RT-qPCR analysis was performed to determine changes in *IFNG* or *TNFA* mRNA expression (*n* = 7, repeated-measures 1-way ANOVA with Šidák correction). (**J** and **K**) Dusp1 was overexpressed in NK92 cells. Experiments are carried out comparing empty vector (EV) control with Dusp1-overexpressing NK92 cells. Flow cytometry–based analysis of extracellular CD107a and intracellular cytokine presence was performed after exposure to K562 cells. Cytokine secretion was measured using ELISA (**K**) (*n* = 6 each, unpaired *t* test for all, except Mann-Whitney for TNF-α dataset; all tests were 2 tailed). Significance was established at *P* < 0.05; asterisks indicate significance between groups. *P < 0.05; **P < 0.01; ***P < 0.001.

To discover pathways connecting obesity and LDL to NK cell function, we took an unbiased approach (Supplemental Figure 2A). We compared the transcriptomic changes induced by LDL in healthy volunteer NK cells to the differentially expressed genes (DEGs) in W/O versus WO/O (cohort 1) participants (Figure 4, A–C, and Supplemental Figure 2). Principal component analyses showed variation in the gene expression count of W/O and WO/O cells (Supplemental Figure 2B) and in vitro LDL-treated versus vehicle control NK cells (Supplemental Figure 2C). 55 DEG were identified in W/O versus WO/O, with 52 upregulated and 3 downregulated (Figure 4A). Pathway enrichment analysis showed significant enrichment of NF-κB signaling, oxidative stress pathways, and protein metabolism (Supplemental Figure 2F). 63 DEGs were seen in LDL-treated NK cells versus control NK cells, with 46 upregulated and 17 downregulated (Figure 4B). Pathway enrichment analysis showed significant enrichment in cytokine-related pathways and lipid metabolism (Supplemental Figure 2G).

To identify genes commonly perturbed in NK cells from AA female individuals W/O and in in vitro LDL-exposed NK cells, DEGs from both datasets were analyzed together (Figure 4C). After taking the intersect, 1 gene was identified to be significantly differentially expressed in both datasets; the gene encoding dual specificity protein phosphatase 1 (*DUSP1*) was highly induced by both obesity and LDL (W/O vs. WO/O, logFC = 2.91, *P* = 0.00002 and control vs. LDL, logFC = 0.67, *P* = 0.0002).

NK cell *DUSP1* gene expression significantly correlated with BMI (*r* = 0.74, *P* = 0.02) and displayed a trending correlation with the ASCVD 10-year risk score (*r* = 0.58, *P* = 0.08) (Supplemental Figure 3, A and B). Furthermore, while no significant correlation could be observed between *DUSP1* expression levels and overall NK cells, we observed a negative association with the cytotoxic NK cell subset and, in turn, a positive association with the proliferative NK cell subset (*r* = –0.67, *P* = 0.04, and *r* = 0.75, *P* = 0.02, respectively; Supplemental Figure 3, C and D). No significant association between LDL concentration and LDL particle number was observed in the participants. The strength of the observed associations increased in participants without lipid-lowering therapy but did not reach significance (Supplemental Figure 3E).

DUSP1 (MKP-1) is the first characterized dual-specific phosphatase able to dephosphorylate and inactivate all three MAPKs (JNK, p38, ERK). As an inducible DUSP family member, it is a negative feedback regulator of NF-κB signaling. Loss of Dusp1 leads to increased expression of LPS-induced TNF-α, IL-6, or IFN-γ of various cell types, highlighting its role in the regulation of inflammation. Furthermore, Dusp1 regulates proliferation, autophagy, metabolic homeostasis, and glucocorticoid signaling (33).

To examine if LDL exposure of healthy volunteer NK cells affected not only *DUSP1* mRNA expression but also its protein expression, we performed flow cytometry analysis of intracellular Dusp1 expression. LDL exposure of healthy volunteer NK cells resulted in a 32.9% ± 10.1% increased expression of Dusp1 protein (*P* = 0.009) (Figure 4D).

Our finding that LDL and obesity highly induced *DUSP1* in NK cells led us to hypothesize that Dusp1 might mediate lipid/obesity-induced NK cell functional changes. To test this hypothesis, we analyzed public NK cell transcriptomic datasets for associations between *DUSP1* expression and NK cell function. *DUSP1* expression decreased by a mean of 18.7% (*P* = 0.27) in NK cells activated with IL-12 (GSE50838) (34), a strong inducer of NK cell cytotoxicity. Additionally, *DUSP1* was downregulated 2-fold (*P* < 0.0001) after NK cell expansion using the Amplification and Expansion System (NKAES) with irradiated K562mbIL21 feeder cells in the presence of IL-2 (GSE128696) (35) (Supplemental Figure 3, C and D).

To assess the role of Dusp1 in NK cell function more directly, we utilized two experimental approaches. First, we utilized pharmacological Dusp1 inhibition on LDL-mediated NK cell functional changes (Figure 4, E–I). Scanning electron microscopy revealed that Dusp1 inhibition rescued LDL-driven changes in NK cell morphology (Figure 4E). These findings could be verified by quantifying F-actin–labeled NK cells that displayed protrusions (Figure 4F). Dusp1 inhibition prevented the LDL-induced reduction in NK cells protrusion; the number of NK cells displaying protrusions was not significantly different with LDL+Dusp1 inhibitor treatment as compared with vehicle control (vehicle control, 62% ± 3.1% vs. LDL, 46% ± 2.7% vs. LDL+Dusp1inh, 54% ± 2.7%). We also found that Dusp1 inhibition rescued LDL-induced reductions of CD107a surface expression (degranulation), intracellular TNF-α expression, and TNF-α secretion (Figure 4, G and H) while not impacting either IFN-γ or TNF-α gene expression (Figure 4I). We then overexpressed Dusp1 in the NK92 NK cell line. Overexpression decreased the expression of CD107a by 30.4% ± 3% and intracellular TNF-α by 25.3% ± 7% (Figure 4, J and K) and inhibited IFN-γ and TNF-α release.

### Dusp1 promotes lysosomal dysfunction in NK cells.

**Figure 5:**
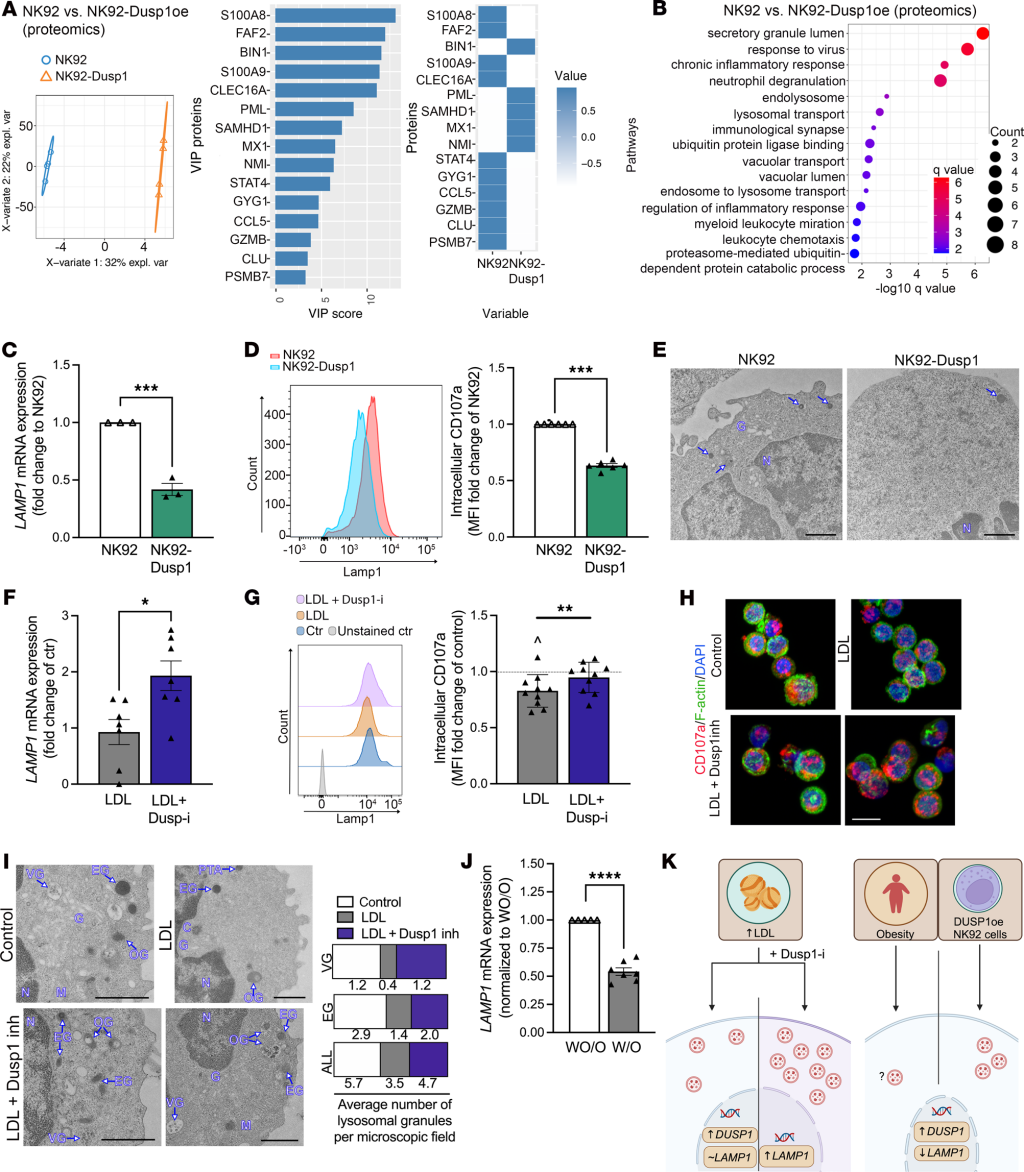
Dusp1 affects lysosome biology. Dusp1 was overexpressed in NK92 cells. The created cell line and its empty vector control were examined to better understand pathways regulated by Dusp1. (**A** and **B**) Proteomics analysis of DUSP1-overexpressing (oe) NK92 cells (*n* = 4 sets). (**A**) Partial least square discriminant analysis (PLSDA) plot of the proteomics data of DUSP1-overexpressing NK92 cells. (**B**) Top 15 gene ontology (GO) terms enriched by proteins that discriminate DUSP1oe NK92cells from NK92cells. Each dot is an enriched GO term labeled on the *y* axis; the size of the dot is scaled to the number of DEGs associated with GO term and is plotted on a negative log_10_ *q* value on the *x* axis. (**C**–**E**) Dusp1oe NK92 cells and their controls were examined for *LAMP1* mRNA expression (*n* = 3, unpaired 2-tailed *t* test), intracellular Lamp-1 protein expression using flow cytometry (*n* = 6, unpaired 2-tailed *t* test), and TEM imaging (*n* = 3 pooled; scale bar: 1 μm). (**F**–**I**) Freshly isolated NK cells were treated with vehicle, LDL, or LDL+Dusp1inhibitor. (**F**) RT-qPCR was performed to determine *LAMP1* mRNA levels (*n* = 7, repeated-measures 1-way ANOVA with Tukey’s correction). (**G**) Flow cytometry was used to quantify intracellular CD107a expression (*n* = 10, repeated-measures 1-way ANOVA with Šidák correction). (**H**) Immunofluorescence analysis of CD107a (red) to determine the presence of intracellular lysosomes, visually confirming flow cytometry results (*n* = 4). Green = F-actin and blue = DAPI. Scale bar: 10 μm. (**I**) TEM analysis of *n* = 3 (pooled). The number of vesicular granules (VG), electron-dense granules (EG), and all granules (VG, EG as well as other granules (OG) was counted per microscopic field and graphed per treatment condition. Representative images are shown. Scale bar: 1 μm. (**J**) RT-qPCR of freshly isolated NK cells of individuals with or without obesity (W/O, *n* = 7 vs. WO/O, *n* = 5) to determine differences in *LAMP1* mRNA expression. (**K**) Graphical summary of the findings in this figure. Significance was established at *P* < 0.05; asterisks indicate significance between groups. *P < 0.05; **P < 0.01; ***P < 0.001; ****P < 0.0001.

To further explore the effect of Dusp1 on NK cells, an unbiased gene expression and proteomics profiling of the Dusp1-overexpressing NK92 cell line compared with the control vector NK92 cell line was performed (Figure 5, A and B, and Supplemental Figure 4, A–C). Differential expression and principal component analyses showed that samples clustered based on the *DUSP1* overexpression status (Supplemental Figure 4A). A large number of upregulated and downregulated genes were observed (Supplemental Figure 4B), and pathway enrichment analysis revealed a variety of significantly changed pathways (Supplemental Figure 4C). Proteomics analysis of Dusp1-overexpressing NK92 cells revealed significant changes and regulation of cytokine-related processes (Figure 5, A and B). For example, S100A8, shown to mediate inflammation in NK cells, and GZMB, which is important for NK cell cytotoxicity, were downregulated in Dusp1-overexpressing cells. Proteomics analysis of Dusp1-overexpressing NK92 cells also showed dysregulation of several biological processes involved in lysosomal presence and function.

To validate these findings and examine the effect of Dusp1 overexpression on lysosomes, RT-qPCR and flow cytometry were performed. Dusp1-overexpressing NK cells displayed 59% reduced *LAMP1* mRNA levels, while *TFEB*, a transcription factor previously reported to regulate *LAMP1* (CD107a) expression (36), was not affected (Figure 5C). There was a 47% reduction in intracellular LAMP1 (CD107a) protein by flow cytometry (Figure 5D), accompanied by reduced lysosomal vesicles in transmission electron microscopy (TEM) (Figure 5E).

To verify if these findings also apply to LDL-treated primary NK cells, a set of similar experiments was performed in the presence or absence of the Dusp1 inhibitor (Figure 5, F–I). Treatment with LDL did not significantly affect *LAMP1* mRNA, but the addition of the Dusp1 inhibitor increased *LAMP1* mRNA compared with LDL exposure alone (Figure 5F). Additionally, we found that intracellular Lamp1 (CD107a) protein expression was decreased after LDL exposure, which could be prevented by the concurrent addition of the Dusp1 inhibitor (Figure 5, G and H). Since lysosomal location and positioning within a cell affects its pH (37) and is crucial for the cytolytic function of cytotoxic T lymphocytes (38), and cytokine secretion is dependent on a variety of lysosomal vesicles (39), we quantified the number of lysosomes and examined vesicle location by TEM (Figure 5I) in a pooled sample of 3 independent experiments. LDL exposure reduced the number and altered the location of lysosomal vesicles of various morphologies (40), supporting intracellular trafficking impairment and potentially explaining reduced cytokine secretion from NK cells with LDL exposure (Figure 5I). Inhibition of Dusp1 ameliorated the effect of LDL, as more lysosomes of all subtypes were found to be closer to the cell periphery overall (Figure 5H).

To determine if a loss in lysosomes was also present in NK cells of individuals W/O, we performed RT-qPCR analysis (Figure 5J) and found that *LAMP1* mRNA expression was 45.8% ± 3% reduced when compared with individuals WO/O, highlighting the similarities between hyperlipidemia and obesity in NK cells (Figure 5K).

### Dusp1 inhibition does not rescue the effect of LDL on lysophagy.

Prior studies have shown a relationship between various lipids, including LDL, and lysosomal damage (41); lysosomal damage has been associated with lysosomal repair processes, autophagy of damaged lysosomes (lysophagy), and/or replenishment in a galactin3/8-dependent manner (42). Interestingly, proteomics analysis of both datasets (LDL vs. control and DUSP1-NK92oe vs. NK92 cells) showed that proteins involved in proteasome-dependent lysophagy were regulated. Therefore, we examined if LDL induced lysophagy and lysosome replacement in a Dusp1-dependent manner (Supplemental Figure 5, A–D) in this set of experiments. While LDL treatment of NK cells initiates ubiquitin labeling of lysosomes, increases ubiquitin as well as galectin-3^+^ NK cells, and decreases galectin-8^+^ NK cells, these effects were Dusp1-independent because the addition of the Dusp1 inhibitor failed to rescue LDL-induced effects. Additionally, TEM images displayed NK cells after LDL exposure that were filled with autophagosomes and autolysosomes. However, the addition of a Dusp1 inhibitor only partially reversed the LDL-induced effect on autophagosome presence or autophagic flux (Supplemental Figure 5, E and F).

### DUSP1 likely promotes lysosome biogenesis via mTOR and TFEB.

**Figure 6:**
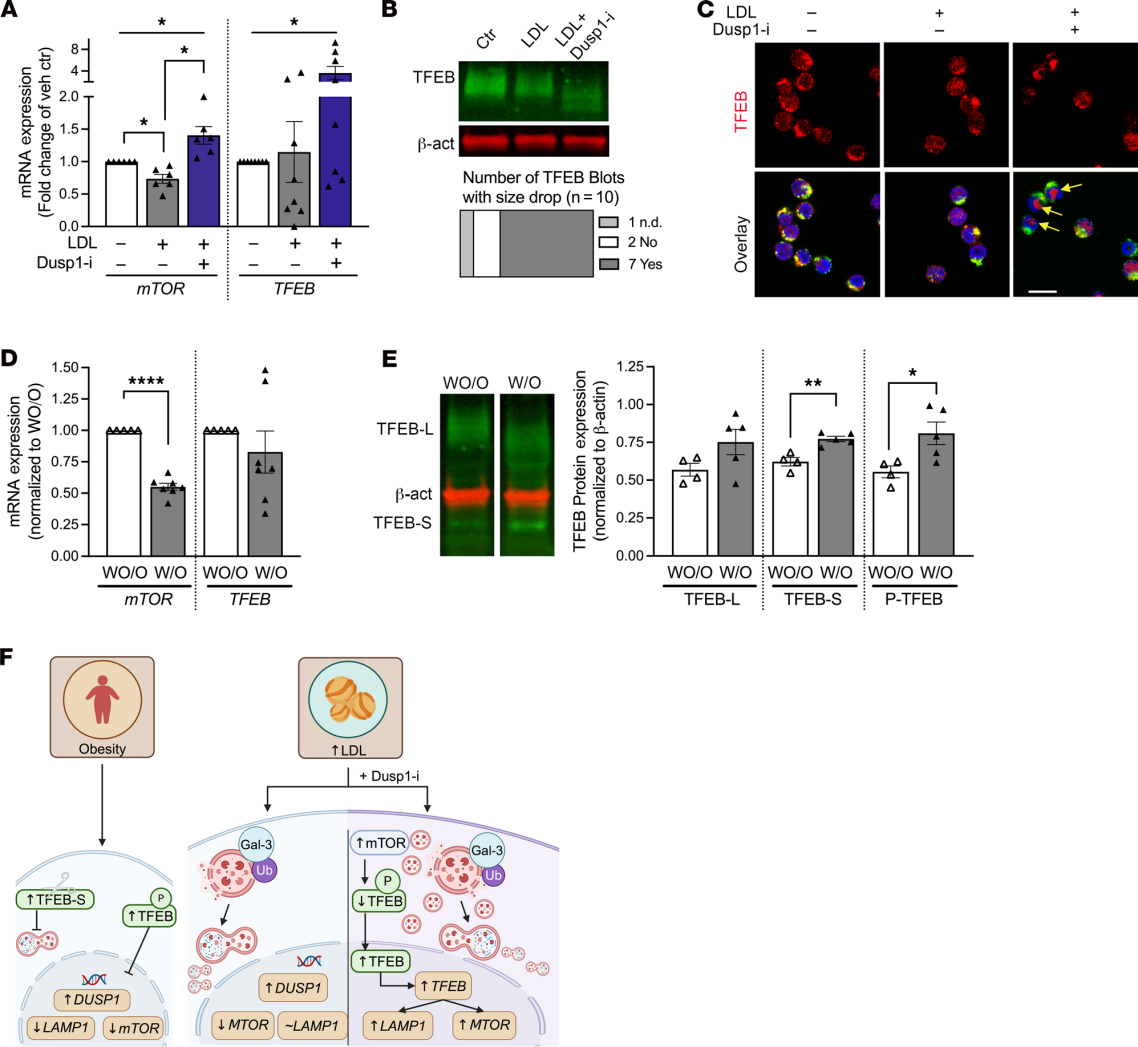
Dusp1 inhibition rescues LDL-induced lysosome depletion via mTOR and TFEB. Freshly isolated primary NK cells were treated with vehicle, LDL, or LDL+Dusp1inhibitor. (**A**) RT-qPCR was performed to determine the affect of LDL and its dependency on Dusp1 on mRNA levels of *mTOR* and *TFEB*, a transcription factor for lysosomal biogenesis (*mTOR*, *n* = 6, repeated-measures 1-way ANOVA with Holm-Šidák correction; *TFEB*, *n* = 8, Friedman’s test with Dunn correction). (**B**) Western Blot analysis of TFEB and β-actin expression. No overall expression differences were observed. Seven of 10 blots displayed a lower TFEB band, indicating decreased TFEB phosphorylation (*n* = 10). (**C**) Visualization of TFEB expression utilizing immunofluorescence of TFEB (red) to the nuclei (blue). Yellow arrows indicate translocalization of TFEB. Lysosomes were labeled in green (*n* = 3). Scale bar: 10 μm. (**D** and **E**) Freshly isolated primary NK cells from individuals with or without obesity (W/O vs. WO/O) were subjected to RT-qPCR and Western blotting analysis. (**D**) RT-qPCR was used to determine potential differences in *MTOR* and *TFEB* mRNA expression in obesity (*n* = 5 WO/O, *n* = 7 W/O; unpaired 2-tailed *t* test for each primer). (**E**) Western blotting was used to examine changes in TFEB expression in NK cells isolated from individuals with or without obesity. (*n* = 4 WO/O, *n* = 5 W/O; unpaired 2-tailed *t* test for each protein). (**F**) Graphical summary of the findings in this figure. Significance was established at *P* < 0.05, comparing individual groups, and is indicated by asterisks. *P < 0.05; **P < 0.01; ****P < 0.0001.

In a last set of in vitro experiments, we sought to determine how Dusp1 inhibition mediates lysosomal replacement in LDL-exposed primary NK cells. Transcription factor EB (TFEB) is a well-known regulator of autophagy and lysosomal biogenesis (43). To initiate lysosomal biogenesis, TFEB is dephosphorylated by mTOR and subsequently translocated to the nucleus. We first utilized RT-qPCR to determine LDL-induced changes in *MTOR* and *TFEB* gene expression levels (Figure 6A). In freshly isolated primary NK cells, LDL treatment was accompanied by a 26.5% ± 0.1% reduction in *MTOR* mRNA expression, which was ameliorated when Dusp1 was simultaneously inhibited. *TFEB* gene expression was unchanged after LDL exposure but significantly increased after LDL+Dusp1inh treatment (LDL, 1.1 ± 0.5–fold change to vehicle control; LDL+Dusp1inh, 3.6 ± 1.2–fold change to vehicle control; Figure 6A). Next, we examined TFEB protein expression by Western blotting (Figure 6B). We did not see overall expression changes between the treatments but a consistent drop in the size of the TFEB band, which indicates dephosphorylation of TFEB in the LDL+Dusp1inh-treated samples. For dephosphorylated TFEB to initiate lysosomal replenishment, a translocation to the nucleus needs to occur. By utilizing immunofluorescence imaging of freshly isolated primary NK cells, we determined that translocation to the nucleus could only be observed in LDL+Dusp1 inhibitor–treated NK cells (Figure 6C), explaining the replenishment of lysosomes (Figure 5, E–G) in LDL+Dusp1 inhibitor–treated NK cells.

To explore the potential importance of the mTOR/TFEB/LAMP1 axis in NK cells isolated from individuals W/O or WO/O obesity, we performed RT-qPCR analysis and Western blotting analysis. RT-qPCR showed a significant 44.9% ± 3% reduction in *MTOR* and gene expression, while *TFEB* gene expression was unchanged (Figure 6D).

Western blotting revealed that NK cells isolated from individuals W/O did not show overall TFEB protein expression differences (WO/O, 0.57 ± 0.04 vs. W/O, 0.75 ± 0.08, *P* = 0.11) but the increased presence of phosphorylated TFEB (WO/O, 0.56 ± 0.04 vs. W/O, 0.81 ± 0.07, *P* = 0.03) and the increased presence of a 31-kDa TFEB splice variant, reported to regulate TFEB function negatively (WO/O, 0.62 ± 0.03 vs. W/O, 0.77 ± 0.02, *P* = 0.002, Figure 6E). These findings suggested dysregulated autophagy and lysosomal biogenesis in NK cells isolated from individuals W/O, with potential roles for mTOR and TFEB in the DUSP1 cascade (Figure 6F).

## Discussion

The associations between aSDoH and obesity are well-documented and represent a major source of disparities in both CVD and cancer morbidity and mortality. However, the underlying mechanisms and subsequent consequences on immune cell function and intracellular signaling are incompletely understood. In this study, we focused on the potential effects of obesity and hyperlipidemia on NK cells in a high-risk population of AA women disproportionately impacted by aSDoH. We aimed to identify common signaling pathways between changes induced by obesity and hyperlipidemia. In exploratory analyses of data from this population, lower SES, higher social isolation, and greater depressive symptoms were associated with a pathological shift in NK cell immunophenotype. NK cells of AA women W/O also displayed reduced degranulation, cytolysis, and cytokine secretion as compared with age-matched AA women WO/O. Among AA individuals not on lipid-lowering therapy, higher LDL was associated with NK cell dysfunction. Accordingly, LDL promoted a dysfunctional NK cell phenotype that recapitulated the immunophenotype observed in NK cells from AA women W/O. Unbiased omics-based analyses identified *DUSP1* as a crucial and common regulator of obesity- and LDL-mediated NK cell dysfunction. This was further supported by the effects of Dusp1 inhibition and overexpression on basal NK cell function and LDL-induced NK cell dysfunction. Together, these results strongly suggest that, for individuals exposed to aSDoH, obesity and hyperlipidemia promote NK cell dysfunction at least partially through pathways initiated by LDL involving Dusp1-mediated regulation of mTOR- and TFEB-facilitated lysosomal biogenesis.

To our knowledge, this is the first study to specifically investigate obesity- and hyperlipidemia-related NK changes in AA women experiencing chronic aSDoH. Prior studies have examined the relationship between aSDoH and NK cells, but these studies have often been done in animal models (44, 45) or potentially confound results by not providing results from minoritized populations or individuals at increased risk for CVD (46, 47). From epidemiologic studies, aSDoH, including neighborhood deprivation (48), lower socioeconomic resources (49), depression (50), and perceived stress (51), are associated with incident obesity, especially among AA women who are disproportionately impacted by aSDoH and subsequent obesity (52). Prior work has shown that obesity negatively affects NK cell phenotype and function (20), as do the experience of aSDoH, e.g., depression (53) and maternal deprivation (54). We observed a switch in NK cell immunophenotype toward a CD56^bright^/CD16^dim/–^ population, which is associated with reduced cytotoxicity. Accordingly, we detected reduced degranulation, surface CD107a expression, and IFN-γ production (17). More work is needed to determine if the NK cell immunophenotypic changes observed in this study may occur in the setting of aSDoH, independent of prevalent obesity.

Despite the existing body of literature supporting obesity- and LDL-related NK dysfunction, the mechanisms by which obesity and LDL together modulate NK biology are still incompletely understood. Lipids are thought to play a key role; NK cells of individuals W/O can undergo metabolic reprogramming due to intracellular lipid enrichment and subsequent mitochondrial dysfunction (17). While we observed obesity-related NK cell dysfunction, our results did not suggest mitochondrial pathogenesis. This difference could be related to differences in sample processing. We performed our experiments on primary NK cells, while other studies have expanded NK cells prior to analysis or utilized NK cells derived from cryopreserved PBMCs (27, 55, 56). By investigating primary NK cells, we were able to link obesity-related NK cell dysfunction to serum LDL levels. This was based on the association of serum LDL levels with NK cell dysfunction in participants W/O as well as pathological changes in NK cell phenotype and function after in vitro exposure to LDL.

One of our central findings is that obesity, as well as LDL, reduced NK cytotoxicity and function through Dusp1. The effects of LDL were only seen in primary NK cells and not in the IL-2–dependent NK-92 cell line, consistent with the observation that IL-2–mediated NK activation can prevent LDL-mediated repression of cytotoxicity (25). Nonetheless, our findings reinforce the role of LDL as a potent NK cell immunomodulator. Moreover, our transcriptomic analysis identifies *DUSP1* as a common factor linking obesity- and LDL-mediated regulation of NK cells. Dusp1 is a potent negative regulator of MAPK-induced NF-κB activation (33), and lipids are reported to induce inflammation through NF-κB activation. Public dataset analysis has demonstrated *DUSP1* expression in specific NK cell subtypes, indicating a potential role in NK biology (57, 58). Therefore, our data suggest that Dusp1 may provide a novel mechanistic link between obesity, LDL, and NK cell dysfunction. Dusp1 can also inhibit mast cell degranulation (59), further supporting a potential role in repressing NK cell degranulation.

Our results may also help to explain the mechanisms underlying obesity-related malignancy risks. NK cells utilize protrusions to recognize and form immunological synapses with tumor cells, thereby facilitating antitumor cytotoxicity. Tumors can evade NK cell–mediated cytotoxicity by reducing protrusion abundance on tumor-infiltrating NK cells (32). LDL reduces the abundance of NK cell protrusions in a Dusp1-dependent manner, which could promote malignancy in individuals W/O and hyperlipidemia. While future investigations should clarify the mechanisms of LDL-driven protrusion loss, our transcriptomic results implicate Rho-GTPases as a potential link. Rho-GTPases mediate cytoskeletal reorganization (60) and are dysregulated by LDL in NK cells. This suggests that Rho-GTPases might regulate LDL-related NK protrusion loss.

Although Dusp1 has not previously been implicated in obesity- or LDL-related immune suppression, it is strongly linked to prevalent obesity (61). Dusp1 is upregulated in the subcutaneous adipose tissue, plasma, and PBMCs of individuals W/O (62). This obesity-induced Dusp1 upregulation is reduced by physical activity (62). *DUSP1* single nucleotide polymorphisms are also associated with obesity-related metabolic complications (63). DUSP1 is also linked to immune cell function in the context of CVD. Specifically, *DUSP1* was the second-highest DEG in monocytes isolated from patients with atherosclerosis compared with those from healthy volunteers (64). However, murine models have yielded contradictory results on the role of DUSP1 in atherogenesis. Transferring myeloid *Dusp1*^–/–^ bone marrow into *Ldlr*^–/–^ mice accelerates atherosclerosis (65), while germline and myeloid *Dusp1* deletion is protective in *ApoE*^–/–^ mice (66). Our findings suggest that these differences may be due partly to differences in LDL receptor expression between murine models, although cell-specific DUSP1 regulation may also play a role (67). Dusp1 and LDL are also reported to regulate each other. *Dusp1*^–/–^ mice have decreased VLDL/LDL levels (68), while modified and oxidized LDL can induce Dusp1 in endothelial cells (69) and monocytes (70). These findings emphasize the crosstalk between Dusp1 and LDL in immune and stromal cells. Our study advances the field by establishing Dusp1 as a central modulator of obesity and LDL-mediated NK dysfunction in at-risk individuals chronically exposed to aSDoH.

Another key finding of our study is the link between LDL, lysophagy, and NK cell dysfunction through Dusp1. We observed that LDL reduced the abundance of intracellular lysosomes in NK cells, which is likely related to the induction of lysophagy, leading to lysosome depletion, which has been described previously (71). LDL has previously been shown to alter lysosomal function and induce lysosomal damage in endothelial cells and macrophages (28, 41), which could also be detected in LDL-treated NK cells in our study. Moreover, in inflammation-induced cardiomyopathy, Dusp1 regulates mitophagy (72), a selective autophagy process similar to lysophagy. In our experiments, Dusp1 inhibition did not fully rescue autophagy/lysophagy but rescued the observed lysosomal depletion by enhancing mRNA levels of mTOR, allowing for TFEB dephosphorylation and subsequent nuclear translocation, which, in turn, induced lysosomal biogenesis. In prior studies, Dusp1 has been shown to impact mTOR in ovarian cancer (73), and TFEB, in turn, has been shown to negatively bind and regulate the *DUSP1* promotor in melanoma (74). In recent years, TFEB has been proposed as a promising target for future therapies in various diseases, including cancer and CVD (75–77). Furthermore, the importance of the mTOR/TFEB axis of immune cells, not including its potential role in NK cells in atherogenesis, has been summarized in a recent review (78), highlighting the importance of our findings as a crucial addition to the existing literature. Additionally, physical activity activated TFEB and TFEB-related processes in an animal model of Alzheimer’s disease (79), while long-term exercise enhanced lysosomal biogenesis in a TFEB-dependent manner in a mouse model (80). Therefore, the Dusp1/mTOR-TFEB axis could provide an innovative therapeutic target, especially for health behavior interventions.

While we have demonstrated what we believe to be a unique pathway in NK cell dysregulation in obesity and hyperlipidemia for a population disproportionately affected by aSDoH, we do acknowledge study limitations. The cross-sectional analyses of human data in our study limit our knowledge of the directionality of the observed associations. Specifically, the aSDoH analyses were exploratory and do not account for potential confounders of the relationship that may affect both SES and NK cells (i.e., chronic disease risk outside of CVD risk, other psychosocial factors such as chronic stress). Additionally, we could not determine if Dusp1 might indeed be regulated by the exposure to chronic stress as an aSDoH and if this precedes obesity development. Larger, diverse longitudinal studies are needed at different geographic sites to determine causality. While the inclusion of only AA women could be seen as a limitation, we believe focusing on a group at highest risk for obesity and related cardiometabolic disorders is foundational to identifying targets for future interventions for a population traditionally underrepresented in biomedical research but most impacted by aSDoH.

In summary, our data implicate Dusp1 as a crucial obesity- and hyperlipidemia-induced factor that may promote CVD and cancer risk through its immunosuppressive effects. To our knowledge, our study is the first to demonstrate a mechanistic link between obesity, LDL, and NK cell function via Dusp1. The study cohort represents a population at the highest risk for obesity but historically underrepresented in biomedical research, increasing the potential impact of our work. Future studies should further examine the expression of Dusp1 in an immune subset–specific manner and will need to clarify the role of chronic environmental and psychosocial stressors in obesity-related NK modulation. Dusp1 may also play a role in these processes, as the *DUSP1* gene harbors binding sites for important transcriptional regulators of stress-related immune dysregulation, like NF-κB and glucocorticoid receptor (81). In the future, Dusp1 may represent a cell-specific therapeutic target or biomarker in at-risk populations. Future work may involve longitudinal studies of Dusp1 levels in diverse populations most affected by CVD and cancer disparities. Additionally, community-informed interventions targeting aSDoH, obesity, and hyperlipidemia might impact Dusp1-driven NK cell dysfunction to reduce CVD or cancer risk by promoting physical activity and other lifestyle changes (62). Ultimately, this study emphasizes the need for the intentional representation of diverse populations in fundamental mechanistic research and the potential insights gleaned from this approach.

## Methods

### Sex as a biological variable.

Our study focuses on AA women from underresourced communities who, in research, are traditionally underrepresented, excluded, and, in the United States, have the highest risk for obesity when compared with men or people of other races and ethnicities.

### Cohort 1.

Participants (*N* = 29) were AA women from the Washington, DC, metropolitan area. Participants who had either no obesity (WO/O, *n* = 15) or had obesity (W/O, *n* = 14) without prevalent CVD were age matched. Obesity was defined as a BMI ≥30 kg/m^2^. Individuals were recruited to the Clinical Center at the NIH and underwent a physical examination, medical history review, and provided blood samples. Whole blood samples underwent flow cytometry–based analysis of blood cells. Additionally, NK cells were isolated, and the NK cell degranulation assay was performed as described below (WO/O, *n* = 15; W/O, *n* = 13, due to 1 participant with insufficient blood draw). Patient demographics for cohort 1 are described in Supplemental Table 1.

### Cohort 2.

Participants (*N* = 60) were AA adults from the Washington, DC, metropolitan area. Participants underwent a physical exam and clinical labs, and serum was biobanked. Participant demographics for cohort 2 are provided in Supplemental Table 2.

### Characterization of whole blood immune cells and NK cell phenotype.

The study participants’ heparinized whole blood (0.5 mL) was prepared as described previously (82), with more details provided in the Supplemental Methods.

### NK cell isolation and treatment.

PBMCs from peripheral blood from study participants or buffy coats from blood bank donors were isolated using SepMate Tubes and Lymphoprep separation media (both from StemCell Technologies) utilizing the density gradient technique per manufacturers’ recommendation within 2 hours of the blood draw. Next, CD3^+^ cells (T cells and NKT cells) were removed via positive selection. According to manufacturer’s protocol, freshly isolated PBMCs were tagged with magnetic CD3 MicroBeads (Miltenyi) and then magnetically separated using LS Columns (Miltenyi). Similarly, the flow through (CD3^–^ PBMCs) was then subjected to a second positive selection step utilizing magnetic CD56 MicroBeads (Miltenyi) to obtain CD3^–^/CD56^+^ NK cells. Isolated NK cells were used after a 30-minute resting period in Aim V media (Gibco). Afterward, cells were counted and prepared for treatment immediately.

### Degranulation assay — NK cell activity.

The potential of naive freshly isolated NK cells to degranulate toward K562 cells was determined using flow cytometry. K562 cells (CCL-243, ATCC) were cultured in RPMI 1640 supplemented with 10% FBS. Prior setup experiments revealed a 1:1 ratio of freshly isolated naive NK cells/K562 cells to be optimal for our experimental conditions. 2.5 × 10^5^ NK cells were treated as described above and in figure legends. After 18 hours of incubation, the treatment was removed, and K562 cells were added for 5 hours to allow NK cell reaction. After 1 hour of treatment, GolgiStop (BD Biosciences) was added, per the manufacturer’s recommendation, to the coculture to inhibit further cytokine secretion and allow maximum detectable intracellular cytokine labeling for flow cytometry. Afterward, cells were centrifuged at 300*g* for 5 minutes, the supernatant was collected for subsequent ELISA cytokine quantification, and extracellular proteins CD3, CD56, and CD107a were stained using anti-CD3-APC/Cy7 (SK7 clone, BioLegend), anti-CD56-BV450 (NCAM16.2 clone, BD Biosciences), and anti-CD107a-PE (H4A3 clone, BioLegend) in flow buffer (1L, PBS pH 7.4 with 500 μL 0.5 M EDTA pH 8.0 and 0.2% BSA) for 20 minutes at 4°C in the dark. Then, the cells were washed twice using a flow buffer, fixed, and permeabilized using BD Cytofix/Cytoperm solution as recommended by the manufacturer (BD Bioscience). Afterward, intracellular cytokines were stained using anti-TNF-α-FITC (Mab11 clone, BioLegend), anti-IFN-γ-APC (4S.B3, BioLegend), anti-Perforin-PE/Cy7 (dG9 clone, BioLegend), anti-GranzymeB-eFluor450 (N4TL33 clone, Invitrogen), and anti-GM-CSF-PerCP/Cy5.5 (BVD2-21C11 clone, BioLegend) for 30 minutes at room temperature in the dark. Cells were then washed and resuspended in flow buffer containing 1% FA to be analyzed using the LSR Fortessa (BD Bioscience). Data were analyzed using FlowJo 10 software. The gating strategy was set using FMO controls in Cell Activator Cocktail–treated (5476, R&D Systems) NK cells. The Cell Activator Cocktail (a mixture of monensin sodium salt, phorbol 12-myristate 13-acetate, and ionomycin calcium salt) was used per the manufacturer’s recommendation at a 1:500 dilution in media.

### RNA sequencing and bioinformatics analysis of NK cells from participants W/O or WO/O as well as control or LDL-exposed NK cells.

RNA sequencing was performed from freshly isolated primary NK cells from participants’ peripheral blood (*n* = 5 each WO/O or W/O). Healthy donor buffy coats treated with vehicle (control) or LDL (*n* = 4 sets). The detailed protocol can be found in the Supplemental Methods.

### Proteomics.

Protein identification was performed by LC-MS/MS analysis and is described in detail in the Supplemental Methods.

### NK92 Dusp1-overexpressing cell line creation.

Dusp1 was expressed in NK92 cells by the Sleeping Beauty transposon system. An empty vector control cell line was created as the corresponding control. Further methodological details are given in the Supplemental Methods.

### Statistics.

All wet lab–derived data were analyzed using PRISM 7.0 (GraphPad) and Microsoft Excel software. The D’Agostino or Shapiro-Wilk test tested each dataset for normality to determine subsequent statistical models. All significance thresholds were designated at *P* < 0.05. Depending on the nature of the dataset, paired analysis was performed. Normally distributed datasets were analyzed using 2-tailed *t* test, paired 2-tailed *t* test, 1-way ANOVA with Dunnett’s correction for multiple comparisons (no pairing), or mixed-effects analysis with Geisser-Greenhouse correction, and Dunn’s correction for multiple comparison analysis (paired). Nonparametric datasets were analyzed using the Mann-Whitney test, the Kruskal-Wallis test (unpaired), or the Friedman’s test (paired) with Dunn’s correction for multiple comparisons. All data are presented as mean ± SEM, and the exact sample size (*n*) is provided in the figure legends.

Multivariable regression analysis was performed using STATA release 12 (StataCorp). Unadjusted and adjusted multivariable linear regression modeling evaluated the relationship between psychosocial measures, clinical parameters, and ex vivo characteristics. *P* values of less than 0.05 were reported as statistically significant. All these analyses were performed in a blinded fashion owing to the nature of the experiment.

### Study approval.

Study approval was obtained from the IRB at the National Heart, Lung, and Blood Institute and NIH in accordance with the principles of the Declaration of Helsinki. The guidelines for good clinical practice and the Belmont Report (National Commission for the Protection of Human Subjects of Biomedical and Behavioral Research) were followed precisely. Participants were enrolled under IRB-approved clinical trials NCT01143454 and NCT00001846. All study participants provided written informed consent, and blood bank donors were deidentified before samples were received.

### Data availability.

All data are available upon reasonable request. Values for all data points in graphs are reported in the Supporting Data Values file.

The RNA-sequencing data were uploaded to the GEO repository (accession GSE278321).

## Author contributions

YB and TMPW conceptualized the study and wrote the initial manuscript. SAK, NRR, KS, and MP analyzed RNA-sequencing data. KS and MP performed proteomics analysis. KS and DMS analyzed publicly available datasets. YB, ASB, CAGH, AS, SJ, LROW, BST, LGM, and JAY performed and analyzed experiments. MRA and EMAP supported the statistical analyses in this manuscript. PKD assisted with all flow cytometry–based assays and analyses. LC and MI created the Dusp1-overexpressing and control NK92 cell lines. VMM, BSC, and TMPW recruited and worked with the community and patients. DSJA, RNR, and RWC provided support and protocols throughout the study. CKEB, CAC, and PD provided crucial support for electron microscopy and fluorescence microscopy experiments or flow cytometry. All authors provided critical feedback and edits to the manuscript.

## Supplementary Material

Supplemental data

Unedited blot and gel images

Supporting data values

## Figures and Tables

**Table 1:**
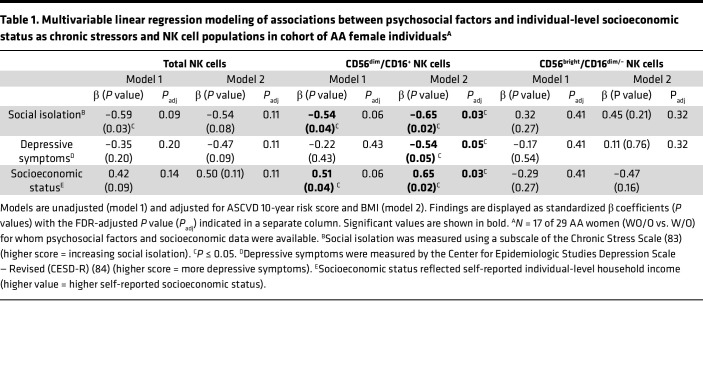
Multivariable linear regression modeling of associations between psychosocial factors and individual-level socioeconomic status as chronic stressors and NK cell populations in cohort of AA female individuals^A^

**Table 2:**
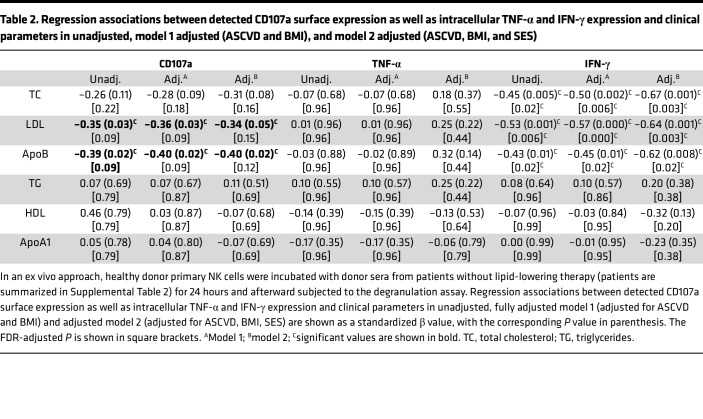
Regression associations between detected CD107a surface expression as well as intracellular TNF-α and IFN-γ expression and clinical parameters in unadjusted, model 1 adjusted (ASCVD and BMI), and model 2 adjusted (ASCVD, BMI, and SES)

## Version 1 12/24/2024

In-Press Preview

## Version 2 01/23/2025

Electronic publication
