# Supplementary material for: Obesity modulates NK cell activity via LDL and DUSP1 signaling for populations with adverse social determinants
Source: JCI Insight. 2024 Dec 24;10(2):e180606. doi: 10.1172/jci.insight.180606 (PMC11790026; doi:10.1172/jci.insight.180606)
Supplement: Supplemental data [file jciinsight-10-180606-s225.pdf]

Obesity modulates NK-cell activity via LDL & DUSP1 signaling for Populations with Adverse Social Determinants

Yvonne Baumer[1], Komudi Singh[2], Abhinav Saurabh[1], Andrew S. Baez[1], Cristhian A. Gutierrez-Huerta[1], Long Chen[3], Muna Igboko[3], Briana S. Turner[1], Josette A. Yeboah[1], Robert N. Reger[3], Lola R. Ortiz-Whittingham[1], Sahil Joshi[1], Marcus R. Andrews[1], Elizabeth M. Aquino Peterson[1], Christopher K.E. Bleck[4], Laurel G. Mendelsohn[1], Valerie M. Mitchell[1], Billy S. Collins[1], Neelam R. Redekar[5], Skyler A. Kuhn[5], Christian A. Combs[6], Mehdi Pirooznia[2], Pradeep K. Dagur[7], David S.J. Allan[3], Daniella Muallem-Schwartz[8], Richard W. Childs[3], Tiffany M. Powell-Wiley[1,9*]

[1] Social Determinants of Obesity and Cardiovascular Risk Laboratory, National Heart Lung and Blood Institute, National Institutes of Health, Bethesda, MD, USA.
[2] Bioinformatics and Computational Core Facility, National Heart Lung and Blood Institute, National Institutes of Health, Bethesda, MD, USA.
[3] Section of Transplantation Immunotherapy, Cellular and Molecular Therapeutics Branch, National Heart Lung and Blood Institute, National Institutes of Health, Bethesda, MD, USA.
[4] Electron Microscopy Core Facility, National Heart, Lung and Blood Institute, National Institutes of Health, Bethesda, MD, USA.
[5] Integrative Data Sciences Section, National Institute of Allergy and Infectious Diseases, National Institutes of Health, Bethesda, MD, USA
[6] Light Microscopy Core, National Heart, Lung and Blood Institute, National Institutes of Health, Bethesda, MD, USA.
[7]Flow Cytometry Core, National Heart, Lung and Blood Institute, National Institutes of Health, Bethesda, MD, USA.
[8] Division of Rheumatology and Clinical Immunology, University of Pittsburgh, Pittsburgh, PA, USA
[9] Intramural Research Program, National Institute on Minority Health and Health Disparities, National Institutes of Health, Bethesda, MD, USA.

Short title: Obesity and adverse SDoH impact NK cell function

*Corresponding author:
Tiffany M. Powell-Wiley, MD, MPH
Chief, Social Determinants of Obesity and Cardiovascular Risk Laboratory,
National Heart Lung and Blood Institute,
Building 10-CRC, Room 5-5332
Bethesda, MD 20892, USA
Telephone: (301) 594-3735
Fax: (301) 402-0888
E-mail: tiffany.powell@nih.gov

Supplementary Tables and Figures:

Supplementary Table 1: Patient demographics of Cohort 1 utilized for NK peripheral blood characterization studies.

	All Participants	WO/O Participants	W/O Participants	p-value between groups	Subgroup with PSES measures available
Sex, Female	29 (100)	15	14	1.00	17 (100)
Ethnicity, African American	29 (100)	15 (100)	14 (100)	1.00	17 (100)
Age, years	54.79 ± 15.8	55.73 ± 18.5	53.79 ± 13.0	0.46	54.02 ± 13.6
Body Mass Index (BMI), kg/m^2	32.10 ± 10.0	24.2 ± 2.2	40.58 ± 7.7	0.000*	33.96 ± 8.9
ASCVD, 10-yr risk score†	6.63 ± 6.3°1	6.41 ± 6.5°2	6.83 ± 6.5	0.73	7.23 ± 5.9
**Medical History:**					
Hypertension	10 (34.4)	2 (14.3)	8 (53.3)	0.03*	7 (41.2)
Diabetes	4 (13.8)	1 (7.1)	3 (20.0)	0.54	2 (11.8)
Hyperlipidemia on Lipid-lowering Rx	6 (20.7)	3 (20.0)	3 (21.4)	1.00	5 (29.4)
**Laboratory Markers:**					
Total Cholesterol (TC), mg/dl	163.7 ± 32.7	172.7 ± 34.9	154.0 ± 31.7	0.15	181.5 ± 25.7
LDL-c, mg/dl	85.4 ± 27.8	85.5 ± 32.3	85.3 ± 23.2	0.91	100.2 ± 24.6
ApoB, mg/dl	67.3 ± 24.7	67.4 ± 29.1	67.2 ± 20.1	0.72	87.7 ± 18.2
Triglycerides (TG), mg/dl	70.7 ± 28.6	73.8 ± 32.5	67.4 ± 24.7	0.87	75.9 ± 21.0
HDL-c, mg/dl	66.9 ± 20.2	75.2 ± 19.6	58.0 ± 17.2	0.008*	66.0 ± 17.6
ApoA1, mg/dl	150.6 ± 31.2	165.4 ± 26.5	134.8 ± 28.6	0.004*	166.3 ± 26.7
**Psychosocial factors and Socioeconomic Status:**					
				Social Isolation ^	0.36 ± 0.7
				Depressive Symptoms &	6.53 ± 6.8
				Socioeconomic Status, household income /$10k#	67.65 ± 30.5

Categorical variables are expressed as the number of participants followed by the percent of the total (%). Continuous variables are expressed as mean ± standard deviation (SD). Significance between the group without or with obesity is indicated with * after Wilcoxon rank-sum testing with the indication of the exact p-value.

†ASCVD 10-year risk score – Atherosclerosis Cardiovascular Disease 10-year risk score

°1n=26, °2n=12 due to 3 individuals missing systolic blood pressure variable

[^]Social Isolation - measured a sub-scale of the Chronic Stress Scale (1)(higher score=increasing social isolation)
[&]Depressive Symptoms were measured by the Center for Epidemiologic Studies Depression Scale - Revised (CESD-R)(2) (higher score=more depressive symptoms)
[#]Socioeconomic Status –self-reported individual-level household income (higher value=higher self-reported socioeconomic status)

Supplementary Table 2: Patient demographics for Cohort 2 used for *ex vivo* serum component studies.

	**Participants (N=60)**
**Sex, Female**	56 (93)
**Ethnicity, African American**	60 (100)
**Age, years**	60.83 ± 10.5
**BMI, kg/m²**	33.00 ± 7.9
**ASCVD, 10-yr risk score**	10.75 ± 8.5
**Hypertension**	38 (63.3)
**Diabetes**	13 (21.7)
**Lipid-lowering therapy**	22 (37)
**TC, mg/dl**	188.98 ± 35.20
**HDL, mg/dl**	66.57 ± 20.58
**LDL, mg/dl**	105.5 ± 33.03
**ApoB, mg/dl**	92.28 ± 24.21
**TG, mg/dl**	84.97 ± 26.43
**HDL, mg/dl**	66.57 ± 20.58
**ApoA1, mg/dl**	169.35 ± 30.29

Categorical variables are expressed as the number of participants followed by the percent of the total (%). Continuous variables are expressed as mean ± standard deviation (SD). (Abbreviations: TC=Total Cholesterol, TG=Triglycerides, BMI=Body Mass Index, ASCVD=Atherosclerotic Cardiovascular Disease)

Supplementary Table 3: In an *ex vivo* approach, healthy donor primary NK cells were incubated with donor serum (patients summarized in Supplementary Table 2) for 24 hours and afterward subjected to the degranulation assay. Regression associations between detected CD107a surface expression as well as intracellular TNFα and IFNγ expression and clinical parameters in unadjusted, fully adjusted model 1 (adjusted for ASCVD and BMI) and adjusted model 2 (adjusted for ASCVD, BMI, SES) shown as standardized ß-value with the corresponding p-value in parenthesis for all study participants independent of status on lipid-lowering therapies. The FDR-adjusted p-value can be found in the square brackets []. (significance was assumed when a $p<0.05$ was reached and is indicated with the asterisk and bolt font; Abbreviations: TC=Total Cholesterol, TG=Triglycerides)

	CD107a			TNFα			IFNγ		
	**Unadj.**	**Adj.[1]**	**Adj.[2]**	**Unadj.**	**Adj.[1]**	**Adj.[2]**	**Unadj.**	**Adj.[1]**	**Adj.[2]**
**TC**	-0.15 (0.26) [0.52]	-0.15 (0.25) [0.50]	-0.20 (0.21) [0.42]	0.06 (0.64) [0.77]	0.02 (0.87) [0.95]	0.07 (0.64) [0.64]	**-0.37 (0.004)* [0.01]***	**-0.34 (0.009)* [0.03]***	**-0.33 (0.03)*** [0.16]
**LDL**	-0.21 (0.11) [0.51]	-0.22 (0.09) [0.45]	-0.22 (0.15) [0.42]	0.12 (0.35) [0.70]	0.08 (0.57) [0.86]	0.14 (0.37) [0.59]	**-0.37 (0.004)* [0.01]***	**-0.35 (0.008)* [0.03]***	-0.29 (0.07) [0.16]
**ApoB**	-0.19 (0.17) [0.51]	-0.21 (0.15) [0.45]	-0.23 (0.15) [0.42]	0.02 (0.89) [0.89]	-0.01 (0.95) [0.95]	0.11 (0.49) [0.59]	**-0.28 (0.04)*** [0.08]	-0.27 (0.05) [0.10]	-0.29 (0.08) [0.16]
**TG**	0.04 (0.73) [0.73]	0.04 (0.77) [0.77]	-0.05 (0.74) [0.89]	0.16 (0.21) [0.70]	0.17 (0.24) [0.86]	**0.33 (0.03)*** [0.18]	-0.04 (0.79) [0.84]	-0.04 (0.77) [0.92]	0.01 (0.93) [0.93]
**HDL**	0.07 (0.59) [0.73]	0.08 (0.53) [0.77]	0.06 (0.69) [0.89]	-0.13 (0.33) [0.70]	-0.13 (0.34) [0.86]	-0.22 (0.14) [0.42]	-0.03 (0.84) [0.84]	-0.00 (0.95) [0.95]	-0.11 (0.45) [0.56]
**ApoA1**	0.05 (0.71) [0.73]	0.06 (0.68) [0.77]	-0.01 (0.93) [0.93]	-0.09 (0.51) [0.77]	0.10 (0.49) [0.86]	-0.13 (0.43) [0.59]	-0.06 (0.69) [0.84]	-0.05 (0.71) [0.92]	-0.12 (0.47) [0.56]

Supplementary Table 4: Primer Information for RT-qPCR

Locus	Primer Sequence (5'- …. -3')	
β-Actin	AGACGCAGGATGGCATGGG	Fwd
	GAGACCTTCAACACCCCAGCC	Rev
ATG5	AGCAACTCTGGATGGGATTG	Fwd
	CACTGCAGAGGTGTTTCCAA	Rev
ATG7	ACCTTGGGGTTGCAATGTAGC	Fwd
	TCCTTGCTGCTTTGGTTTCT	Rev
CTSC	TGCCAACTGCACCTATCTTG	Fwd
	AGGCAGTTCCCACCTTCTTT	Rev
CTSG	TCCTGGTGCGAGAAGACTTT	Fwd
	TGCCTATCCCTCTGCACTCT	Rev
IFNg	GCGAAAAAAGGAGTCAGATGC	Fwd
	CAAACCGGCAGTAACTGGAT	Rev
LAMP1	CTTCAGCAGGGGAGAGACAC	Fwd
	TCTCTGGCGTCAGGAAGAAT	Rev
LYST	CCTTTTAGCAGTTCACCCTCCTA	Fwd
	CACTGCTGGAATGCCTCAGGTA	Rev
mTOR C1	CTTACCTGTTTGCCGTCCAT	Fwd
	CACACACTCCAGGAGCTCAA	Rev
NPC1	CTATGGGAAGTGCGGTGTTT	Fwd
	AGATCCCCATAACCCTTTGG	Rev
NPC2	CATTCCTGAGCCTGATGGTT	Fwd
	AGCACCTCCTCTTCAACGAA	Rev
SLC38A9	CTTTAAGGCTGTTCGCTTGG	Fwd
	CACGAGCCAGGTAGCCTAAG	Rev
TFEB	GCAAGCTCAGGCTGGGAG	Fwd
	TATTGATGGGCCCGGGTGGG	Rev
TNFa	AGCCCATGTTGTAGCAAACC	Fwd
	GGAAGACCCCTCCCAGATAG	Rev

Supplementary Figures

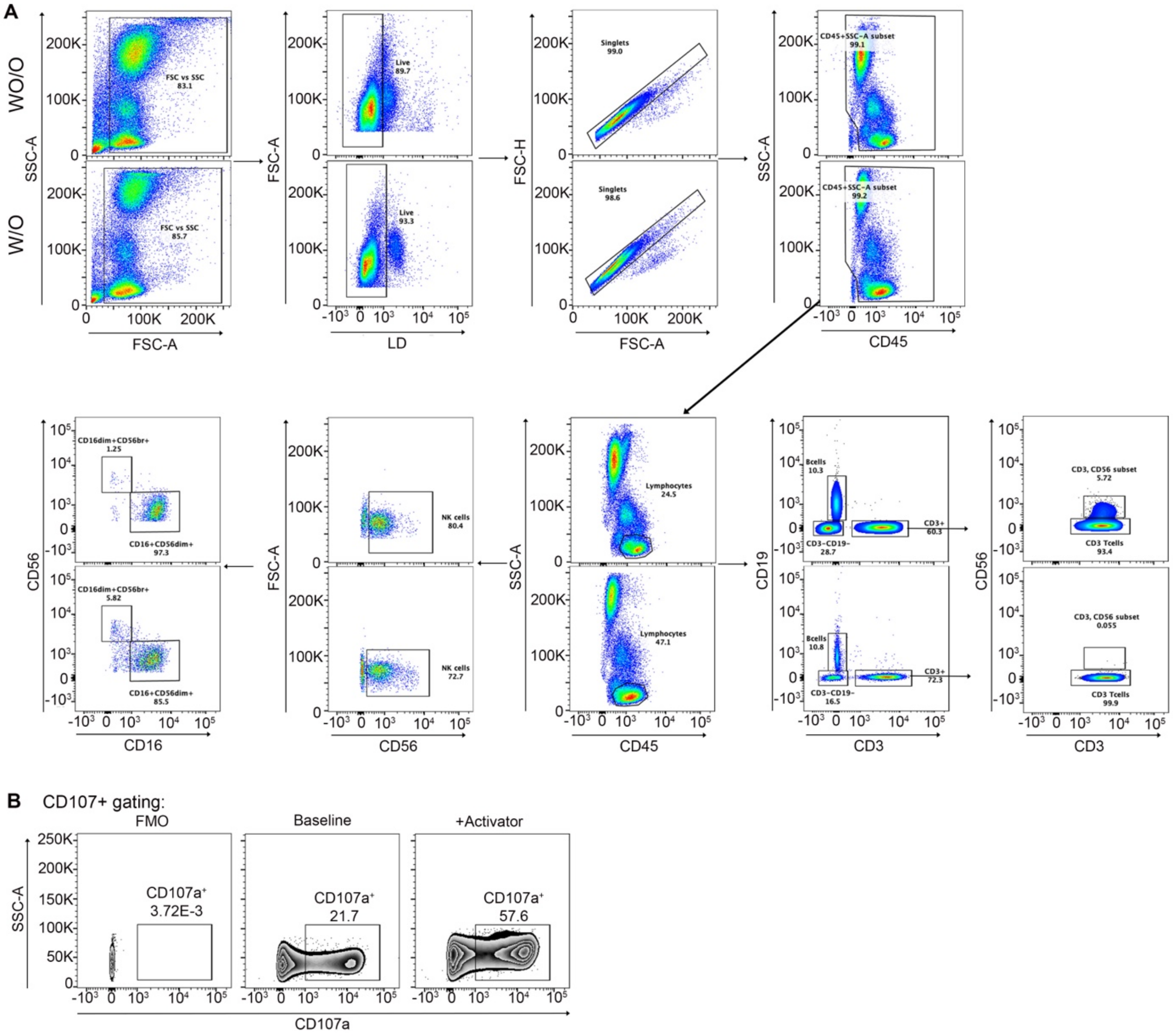

Supplementary Figure 1: NK cells from individuals with obesity displayed altered intracellular cytokine expression.

(**A**) Gating scheme for Figure 1A/B displays one representative sample of a WO/O and a W/O study participant. (**B**) Gating scheme with FMO control for CD107a expression.

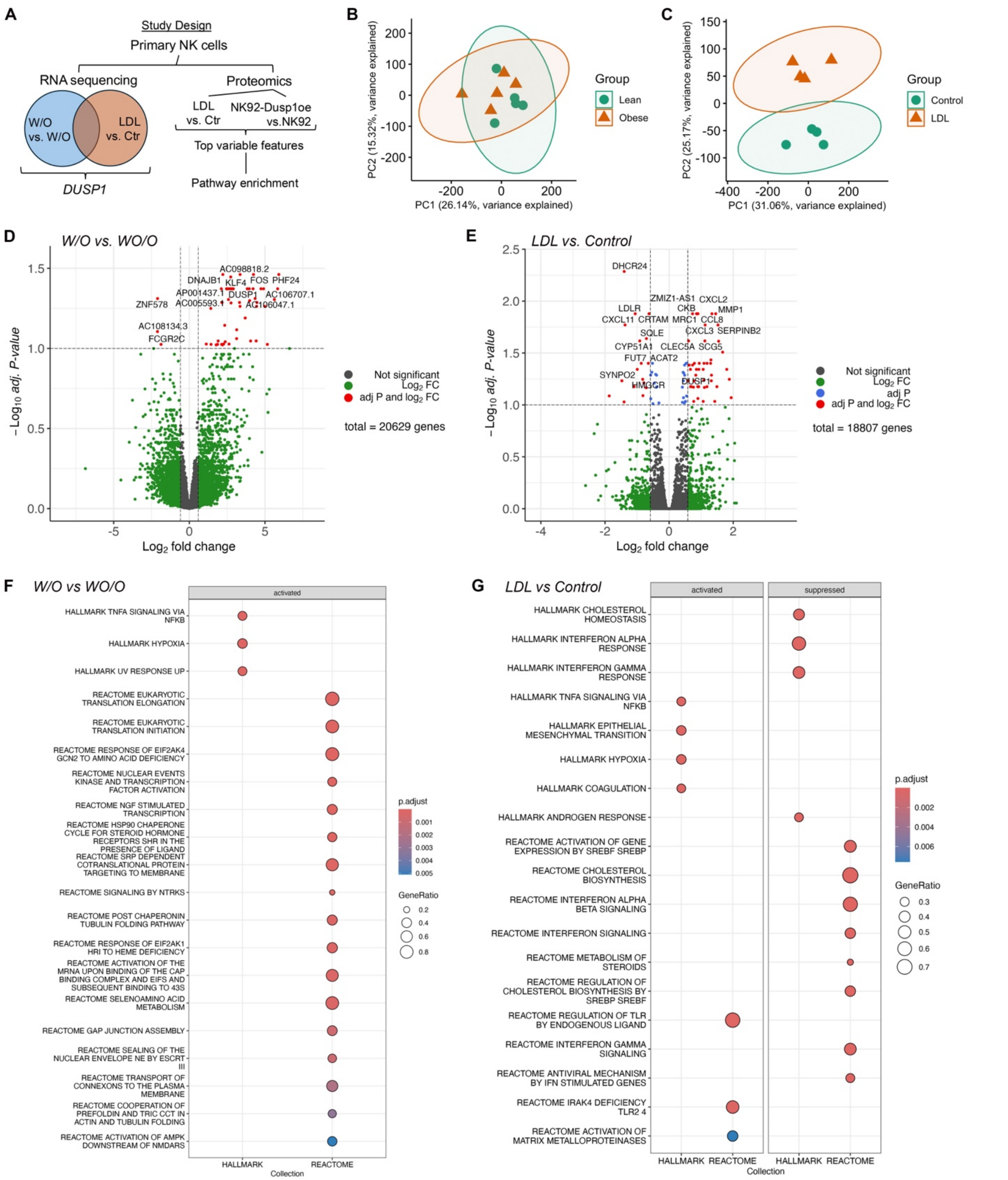

Supplementary Figure 2: Two RNA sequencing datasets derived from NK cells either isolated from individuals with or without obesity or treated with LDL or vehicle control. (**A**) A schema of the unbiased omics analysis was undertaken in this study. (**B-G**) Freshly isolated primary NK cells from 1) either age-matched AA women with (W/O) or without (WO/O) obesity (dataset 1: n=5 each group) or 2) healthy donors treated with LDL or vehicle

control overnight (dataset 2: n=4) were subjected to RNA sequencing analysis. (**B/C**) Principal component analysis (PCA) computed from gene expression data identified in the W/O and WO/O (**B**) or LDL and control (**C**) datasets. Each dot represents a sample from the indicated group. (**D/E**) Volcano plot of genes profiled in W/O vs. WO/O (**D**) or LDL vs. control (**E**) highlights the statistically significant genes (adjusted p-value<=0.1 and FC>=1.5) in red. Genes that meet the adjusted p-value criteria but not the fold change criteria are displayed in blue, while genes that meet the fold change criteria but not the adjusted p-value criteria are shown in green. (**F/G**) Pathways significantly enriched by the differentially expressed genes (adjusted p-value<0.01; absolute normalized enrichment score >2.0) that were concordantly upregulated (or activated) or downregulated (or suppressed) in W/O vs. WO/O (**F**) and LDL vs. control datasets (**G**) comparisons. Each dot is a pathway labeled on the y-axis, and the pathway database is listed on the x-axis. The size of the dot is scaled to the number of concordant DE genes overlapping with the pathway, and the color of the dot is scaled to the adjusted p-value.

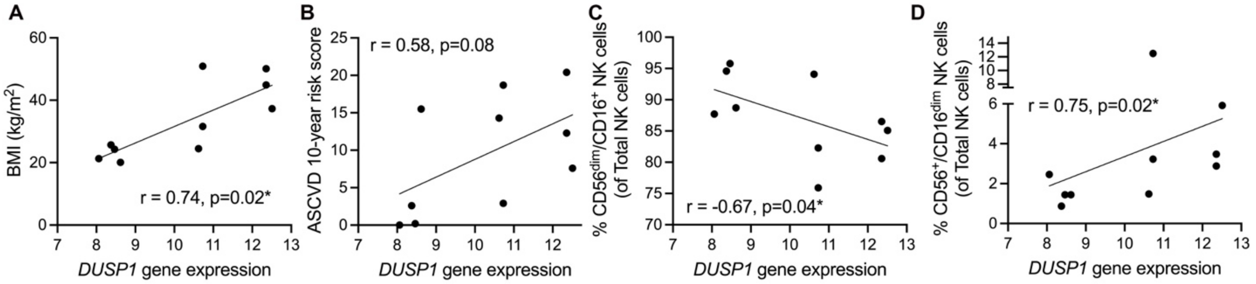

E

	All Participants (n=10)		No Lipid-lowering Therapy (n=6)	
	LDL-c	LDL-p	LDL-c	LDL-p
Scaled DUSP1 gene expression	0.16 (0.66)	0.17 (0.63)	0.55 (0.25)	0.55 (0.25)

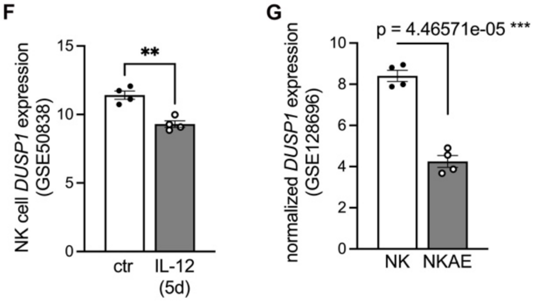

Supplementary Figure 3: Identifying Dusp1 as a commonly regulated gene of potentially crucial importance for NK cell function. *DUSP1* gene expression levels, as determined by scaled *DUSP1* expression from RNA sequencing analysis, were utilized for Spearman correlations with various variables available for the study participants. Data are presented as Spearman correlation coefficient (r) and the corresponding p-value in brackets (n=10, n=6 for participants without lipid-lowering therapy). *DUSP1* gene expression levels were correlated with the study participants' BMI (**A**), ASCVD 10-year risk score (**B**), and proportions of cytotoxic ($CD56^{dim}/CD16^{hi}$) as well as proliferative ($CD56^{hi}/CD16^{dim}$) NK cells (**C/D**). (**E**) *DUSP1* gene expression levels were subjected to Spearman correlation against LDL-c (concentration) and LDL-p (particle number). (**F/G**) Two publicly available datasets on IL-2 treated activated NK cells (GSE50838) as well as activated/expanded NK cells (NKAE, GSE128696) were analyzed for potential changes in *DUSP1* expression to provide evidence that *DUSP1* gene expression is affected when NK cell activity is altered (n=4 in each dataset). (Significance was established when a p-value < 0.05 was present comparing individual groups with the data-appropriate test; *indicates significance between groups; F/G: 2-tailed Mann-Whitney test)

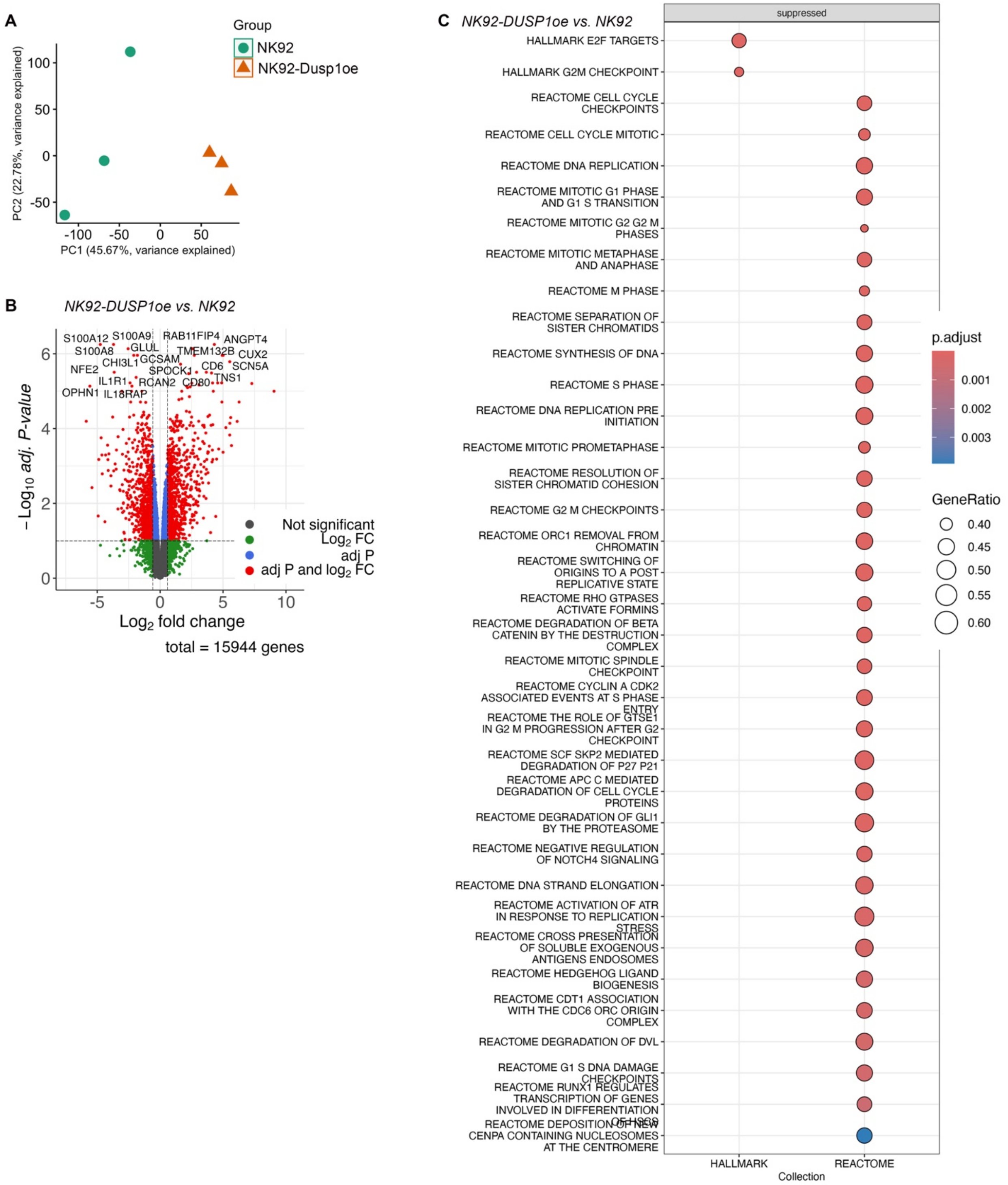

Supplementary Figure 4: Identifying Dusp1 as a commonly regulated gene of potentially crucial importance for NK cell function. Dusp1 was overexpressed in NK92 cells, a well-accepted NK cell line. The created cell line and its control were examined to better understand pathways regulated by Dusp1 (n=3 each). (**A**) Principal component analysis (PCA) shows the variation in the gene expression data of DUSP1 overexpressing (oe) NK92 cells and their respective empty vector control. Each dot represents a sample from the indicated group. (**B**)

Volcano plot of genes profiled in DUSP1 overexpressing (oe) NK92 cells and their respective empty vector control NK92 cells highlights the statistically significant (adjusted p-value <= 0.1 and FC >= 1.5) genes as red. (**C**) Significantly enriched pathways (adjusted p-value <0.01; absolute normalized enrichment score > 2.0) are displayed. Each dot is a pathway labeled on the y-axis and pathway database is listed on the x-axis. The size of the dot is scaled to the number of concordant DE genes overlapping with the pathway and color of the dot is scaled to the adjusted p-value (Significance was established when a p-value < 0.05 was present comparing individual groups; *indicates significance between groups)

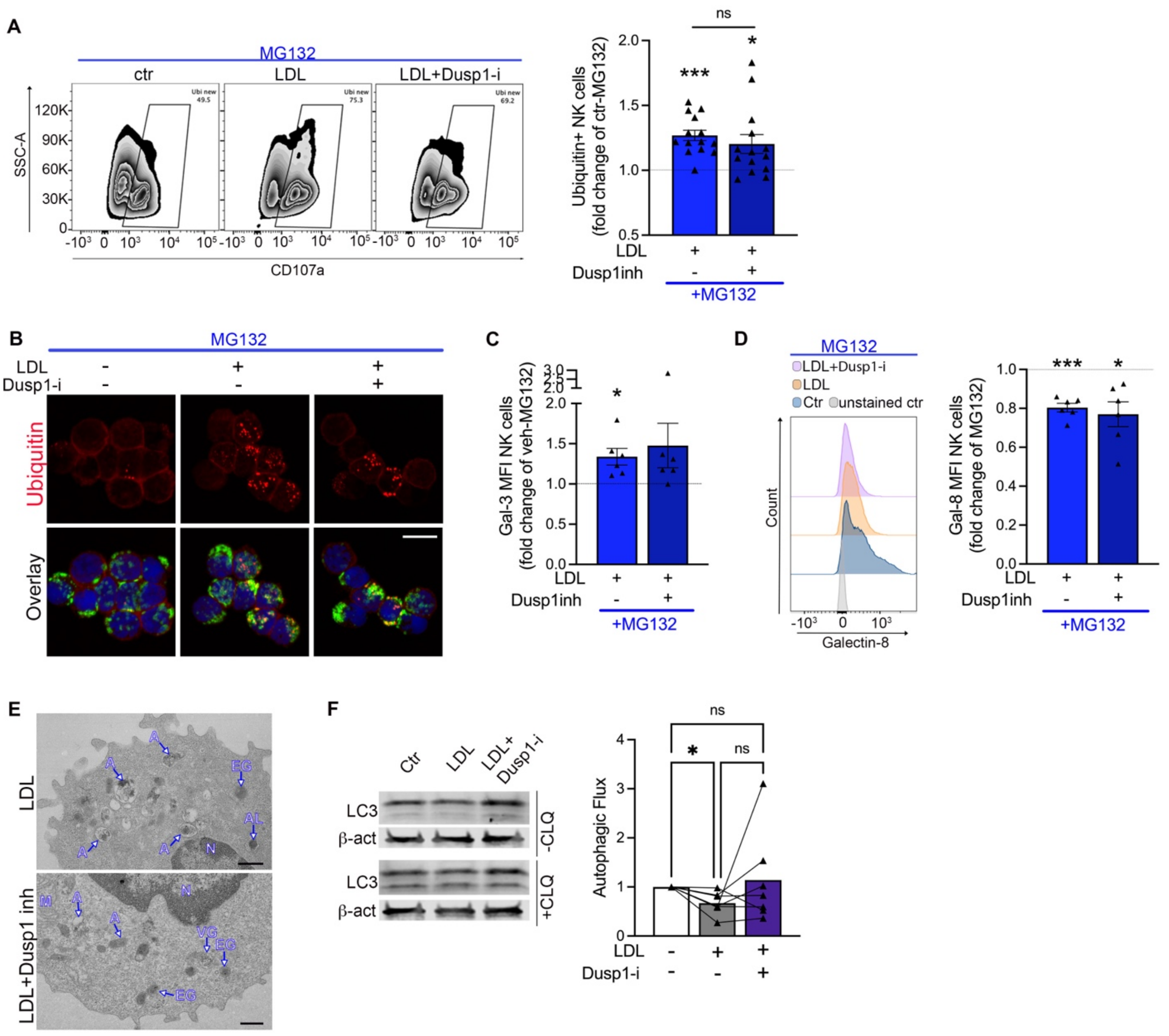

Supplementary Figure 5: Dusp1 inhibition does not rescue LDL-induced lysophagy. Freshly isolated primary NK cells were treated with vehicle, LDL, or LDL+Dusp1inhibitor. Some experiments needed to be performed in the presence of MG-132, a proteasome inhibitor. (**A**) Flow cytometry was used to determine changes in ubiquitin expression. (n=10, RM-One-Way Anova with Holm-Sidak correction) (**B**) Immunofluorescence Imaging of NK cells stained for ubiquitin in red, CD107a in green, and nuclei in blue. Co-localization of CD107a and ubiquitin (yellow) indicate ubiquitin-tagged and, therefore, damaged lysosomes (scalebar 10µm). (n=4) (**C/D**) Flow cytometry-based analysis of galectin-3 or 8 expression. (C: n=6, Friedman test with Dunn's correction; D: n=6, RM-One Way ANOVA with Holm-Sidak correction) (**E**) TEM images of LDL and LDL+Dusp1 inhibitor treated NK cells prepared from 3 independent experiments pooled for TEM preparation after sample fixation. The presence of autolysosomes (AL) and autophagosomes (AP) is indicated, as well as electron-dense vesicles (EG) and vesicular granules (VG). (scalebar: 500nm) (**F**) Western blot analysis of LC3 and beta-actin of

NK cells treated with vehicle control, LDL, and LDL+Dusp1 inhibitor in the presence or absence of chloroquine (CLQ) allowed for quantification of autophagic flux. (n=7, Friedman Test with Dunn's correction)

Supplementary Methods:

Characterization of whole blood immune cells and NK cell phenotype

Heparinized whole blood (0.5ml) from the study participants was prepared as described previously (Gating Scheme provided in Supplemental Figure 1) (3). Immune cells were characterized using the following antibody panel, anti-CD3-PECy7 (UCHT1 clone, BioLegend), anti-CD14-APC (61D3 clone, BioLegend), anti-CD15-BV786 (HI98 clone, BD Biosciences), anti-CD16-BUV395 (3G8 clone, BD Biosciences), anti-CD19-BV650 (SJ25C1 clone, BD Biosciences), anti-CD42b-BV421 (HIP1 clone, BioLegend), anti-CD45-PerCP/Cy5.5 (2D1 clone, BD Biosciences), anti-CD56-FITC (NCAM16.2 clone, BD Biosciences), anti-CD193-APC/Cy7 (5E8 clone, BioLegend), and anti-CD203c-PE (NP4D6 clone, BioLegend). Afterward, cells were thoroughly washed twice using a flow buffer and fixed in a flow buffer containing 1% PFA. All samples were analyzed using the LSR Fortessa (BD Bioscience, USA). Results were obtained using FlowJo10 software (FlowJo LLC, USA) with the gating scheme described previously (3).

Treatments and substances

Isolated NK cells were treated overnight (18h) at 37°C/5% CO_2 in AimV media (Gibco, USA) with or without the addition of 25 - 500µg/ml (2.5 - 50mg/dl) low-density lipoprotein (LDL) (cell culture grade, Alfa Aesar or Athens Research, USA). The concentrations of LDL tested were chosen from the previous literature (4-6) as well as from peer-reviewed manuscripts published by our own group (7-10). The higher end of the chosen concentrations resemble physiological LDL levels with 50mg/dL to be close to pathophysiological lives, as described in the 2023 ACC guidelines (11).
The Dusp1 inhibitor (Millipore Sigma, USA, CAS 15982-84-0) was used at 5µM. The MG132 inhibitor (Millipore Sigma, USA) was used at 500nM. Chloroquine was used at 20µM (Tocris, USA).

Ex vivo experimental setup to determine the impact of patient serum on NK cell function

NK cells were isolated as described in the main manuscript. $2x10^5$ NK cells were treated overnight with 10% patient serum (derived and biobanked from Red Top serum tubes without anti-coagulant at their Clinical Center visit) in Aim V media for 18h. Afterward, the NK cell degranulation assay was performed as described above. Afterward, the results obtained per study participant were utilized in statistical models to determine potential significant correlations with clinical markers detected at the participants' visit date. Due to the nature of this experimental setup, all results are obtained in a blinded fashion.

Cytokine ELISA

The two cytokines, IFNγ and TNFα , were detected from the cell culture supernatants of the degranulation assays utilizing the respective R&D DuoSet (R&D Systems, USA) ELISAs per the manufacturers' recommendation.

Scanning Electron Microscopy (SEM) and Transmission Electron Microscopy (TEM)

NK cells were treated as indicated and immediately fixed in 2.5% glutaraldehyde (0.1M calcium chloride, 0.1M sodium cacodylate buffer, pH 7.2). Samples were prepared on 0.1µm filters as described previously.(3, 8, 12) Gold/palladium sputter-coated NK cells were imaged using the

Hitachi S-3400N1 SEM at 7.5keV and the JOEL JEM1400 transmission electron microscope (NHLBI Electron Microscopy Core).

Flow Cytometry analysis
NK cells were treated as indicated, centrifuged at 300 x g for 5 min, and the supernatant discarded. Next, NK cells were washed twice using flow buffer, fixed, and permeabilized using BD Cytofix/Cytoperm solution as recommended by the manufacturer (BD Bioscience, USA). Subsequently, NK cells were incubated with the appropriate antibody cocktail for 30 min at RT in the dark.
To determine Dusp1 expression levels the following antibody panel was used: anti-Lamp1-AF488 (clone H4A3, BioLegend, USA) and anti-Dusp1-AF594 (clone E6, Santa Cruz Biotechnologies).
To determine galectin-3 or galectin-8 expression the following: intracellular protein and lysosomes were stained using anti-Lamp1-BV421 (clone H4A3, BioLegend, USA), anti-Galectin-3-AF488 (clone B2C10, Santa Cruz Biotechnologies, USA), and galectin-8-AF790 (clone C-8, Santa Cruz Biotechnologies, USA)
After antibody incubation, cells were washed and resuspended in flow buffer with 1% PFA. Flow cytometry was performed using the LSR Fortessa (BD Bioscience, USA), and data were analyzed using FlowJo 10 software (FlowJo LLC, USA).

Immunofluorescence
Cells were incubated as indicated, centrifuged at 300 x g for 5 min, and the supernatant discarded. Subsequently, cells were fixed and permeabilized using the BD Cytofix/Cytoperm solution as recommended by the manufacturer (BD Bioscience, USA). Afterward, cells were washed and incubated with the antibody staining cocktails for 1 hour at RT in the dark.
F-actin was labeled using Phalloidin-AF488 (ThermoFisher, USA), while lysosomes were labeled using anti-Lamp1-AF594 (clone H4A3, Santa Cruz Biotechnologies, USA). Additionally, nuclei were labeled using a DAPI solution. Ubiquitin was stained using anti-ubiquitin-AF594 (clone P4D1, Santa Cruz Biotechnologies, USA), while lysosomes were labeled using anti-Lamp1-AF488 (clone H4A3, Santa Cruz Biotechnologies, USA), and nuclei visualized using DAPI solution.
To image TFEB, a 3-step staining process needed to be employed. First, after fixation and permeabilization, TFEB was labeled using anti-TFEB (#4240, Cell Signaling, USA) at a 1:100 dilution in Permeabilization Wash Buffer (BD Biosciences, USA) overnight. The next day, the cells were washed and incubated for 1 hour in the dark with goat anti-rabbit cy3 secondary antibody (#ab6939, Abcam) at a 1:500 dilution. In the third and final step, lysosomes were labeled with anti-Lamp1-AF488 (clone H4A3, Santa Cruz Biotechnologies, USA) at a 1:50 dilution for 1 hour in the dark in the presence of DAPI.
Confocal imaging was performed using both a Zeiss 880 and a Zeiss 780 microscope with a 63x Plan-Apochromat (1.4 NA) objective. The following excitation and emission parameters were used: DAPI (405nm excitation, 411-480nm emission), AF488 (488nm excitation, 490-562nm emission), AF594 (594nm excitation, 603-721nm emission), and Cy3 (561nm excitation, 566-700nm emission). Lateral pixel sizes were 70-120nm with a 0.2-micron interslice step for z-stack imaging. Multi-color images were collected sequentially with line averages of 2 or 4.

Western Blotting

After NK cell incubation, NK cells were lysed using RIPA buffer with protease/phosphatase inhibitors, sonicated, and centrifuged at 5000g for 5 minutes at 4°C. Protein concentration was determined using the BCA assay. Equal amounts of protein per sample (10 - 25μg) were separated using SDS-PAGE and transferred to a nitrocellulose membrane. The membranes were blocked using Intercept TBS Blocking Buffer (Licor, USA) for 1 hour at room temperature and subsequently incubated in primary antibody overnight at 4°C. The following day, the membranes were washed 3 times using 0.1% TBS-Tween-20 and incubated with appropriate secondary antibodies (Licor, USA) for 1 hour at room temperature in the dark. Subsequently, blots were thoroughly washed using 0.1% TBS-Tween-20 and imaged using a Li-Cor Odyssey imaging system. Western blot analysis was performed using FIJI and Excel software.
All antibodies were diluted in 1:1 Intercept TBS Blocking Buffer: 0.1% TBS-Tween-20: beta-actin (1:10,000; cat# 20536-1AP, ProteinTech, USA); NFκB (clone D14E12), TFEB (cat#4240), Phospho-TFEB (Ser211) (clone 59S8N), p62/SQSTM1 (clone D1Q5S) all 1:1,000 dilution and obtained from Cell Signaling Technologies (USA). LC3 antibody was obtained from Millipore Sigma (#L7543) and used at a 1:1,000 dilution.

RT-qPCR for mRNA expression analysis

RT-qPCR was performed as described previously (13). Briefly, after indicated treatments, NK cells were lysed in RLT lysis buffer with beta-mercaptoethanol, and RNA was isolated using the RNAeasy Mini Kit per the manufacturer's recommendations (#74104, Qiagen, USA). cDNA was obtained using the iScript Reverse Transcription Supermix (BioRad Laboratories, USA), and RT-qPCR was completed using a Roche Lightcycler 96 (Roche Molecular Systems Inc, USA).
A melting curve at the end of each RT-qPCR run ensured the quality of the primer pairs used. All primer information can be found in Supplementary Table 4.

Proteomics

Protein extraction and digestion: Proteins from each group of samples are being quantified using the BCA assay Kit. 20μg of protein was denatured in 8M Urea,25mM TEAB buffer followed by reduction (DTT) and alkylation (iodoacetamide) and then the alkylated proteins were diluted to <1M Urea buffer using 25mM TEAB, pH 8 buffer before performing Trypsin digestion overnight. Each sample was digested overnight using the Trypsin enzyme (enzyme-to-substrate ratio of 1:12.5) to get the peptide solution. Trypsin cleaves proteins at lysine and arginine amino acid residues, yielding tryptic peptides. The samples were acidified the next day using formic acid to bring pH<3 to stop the trypsin activity and were processed through Zip-Tip using C18 tips (Millipore) to clean up and concentrate the peptides before going through mass spec analysis.

Liquid Chromatography – Tandem MS Analysis: Protein identification by LC-MS/MS analysis of peptides was performed using an Orbitrap Fusion Lumos Tribid mass spectrometer (Thermo Fisher Scientific, San Jose, CA) interfaced with an Ultimate 3000 Nano-HPLC apparatus (Thermo Fisher Scientific, San Jose, CA). Peptides were fractionated by EASY-Spray PepMAP RPLC C18 column (2m, 100A, 75 m x 50cm) using a 120-min linear gradient of 5-35% ACN in 0.1% FA at a flow rate of 300 nl/min. The instrument was operated in data-dependent acquisition mode (DDA) using an FT mass analyzer for one survey MS scan on selecting precursor ions followed by 3-second data-dependent HCD-MS/MS scans for precursor peptides with 2-7 charged ions above a threshold ion count of 10,000 with normalized collision energy of 37%. Survey scans of peptide precursors

from 300 to 2000 m/z were performed at 120k resolution, and MS/MS scans were acquired at 50,000 resolution with a mass range of m/z 100-2000.

Protein Identification and Data Analysis: All MS and MS/MS raw spectra from each set were processed and searched using the Sequest HT algorithm within the Proteome Discoverer 2.2 (PD2.2 software, Thermo Scientific). The settings for precursor mass tolerance were set at 12 ppm, fragment ion mass tolerance to 0.05 Da, trypsin enzyme with 2 mis cleavages with carbamidomethylation of cysteine as fixed modifications, deamidation of glutamine and asparagine, oxidation of methionine as variable modifications. The Human sequence database from Swiss Sprot was used for the database search. Identified peptides were filtered for a maximum of 1% FDR using the Percolator algorithm in PD 2.2 along with additional peptide confidence set to medium. The final lists of protein identification/quantitation were filtered by PD 2.2 with at least 2 unique peptides per protein identified with medium confidence. For the quantitation, a label-free approach has been used, where the area under the curve for the precursor ions is used to calculate the relative fold change between different peptide ions.

<u>RNA Sequencing and bioinformatics analysis of NK cells from participants with or without obesity as well as control or LDL-exposed NK cells</u>

RNA sequencing was performed from freshly isolated primary NK cells isolated as described above from participants' peripheral blood (n=5 each lean or obese). Healthy donor buffy coats treated with vehicle (control) or LDL (n=4 sets). After isolation and/or treatment, NK cells were lysed in Trizol (15596026, Thermo Fisher Scientific, USA). RNA extraction was performed using the RNeasy Mini Kit (74104, Qiagen, USA) per the manufacturers' recommendations. An additional step of DNase I treatment was performed, and cells were washed twice thereafter. Concentration was determined using a NanoDrop Spectrophotometer. Libraries were prepared using the Takara SMART V4 kit (Takara Bio, USA) as well as Nextera XT DNA Library Preparation Kit (Illumina, USA), and were sequenced on Illumina Novaseq for paired-end 100bps using standard Illumina sequencing protocol.

Raw base calls from the Illumina NovaSeq were demultiplexed and converted into FastQ file format using bcl2fastq. The raw FastQ files were processed using version 1.9.2 of the OpenOmics/RNA-seek pipeline (14). This pipeline is composed of a comprehensive set of tools for processing RNA-sequencing data. The pipeline includes an extensive set of quality control tools to calculate a variety of different sequencing and library quality-control metrics. Briefly, the quality of each sample was assessed using FastQC v0.11.9 (15), Preseq v2.0.3 (16), Picard tools v2.17.11 (17), FastQ Screen v0.9.3 (18), Kraken2 v2.0.8 (19), QualiMap (20), and RSeQC v2.6.4 (21). The sequencing adapter and low-quality base pairs were trimmed using Cutadapt v1.18 (22). The trimmed reads were aligned against a GENCODE (23) primary assembly release of GRCh38 using the splice-aware aligner STAR version 2.7.6a in two-pass basic mode (24). Gene expression levels were quantified with RSEM v1.3.3 (25) using release 30 of GENCODE's comprehensive gene annotation.

Low-count genes were filtered from the raw count matrix using the filterByExpr function from the R package edgeR v.3.36.0 (26). The filtered raw count matrix was used to estimate normalization scaling factors using the calcNormFactors function from edgeR. For the LDL comparison, donor

information was included as a covariate in the model to account for paired samples originating from the same donor. Heatmaps and PCA plots of the trimmed mean of M-values (TMM) scaled, log2 count per million (CPM) normalized counts were visualized using the R packages pheatmap v1.0.12 (27) and ggpubr v0.4.0 (28). Differential expression analysis was performed using the R package limma v.3.50.1 (29) with the following limma functions: lmFit, contrasts.fit, and eBayes. The resulting p-values of the tested genes were adjusted for multiple testing using the Benjamini-Hochberg method. Genes with an adjusted p-value ≤ 0.1 and an absolute fold-change ≥ 1.5 were considered differentially expressed. Volcano plots were created using the R package EnhancedVolcano v.1.12.0 (30).

A pre-rank score suitable for gene set enrichment analysis (GSEA) was calculated using the following formula: $-1 * \left(log_{10}(pvalue) * (foldchange)\right)$. GSEA was performed on the sorted pre-ranked gene list against the Hallmark and Reactome pathway collections using the universal GSEA function from the R packages clusterProfiler v.4.2.2 (31) and msigdbr v.7.5.1 (32). The resulting p-values were adjusted for multiple testing using the Benjamini-Hochberg method. Pathways with an adjusted p-value ≤ 0.01 and an absolute normalized enrichment score (NES) ≥ 2.0 were considered significantly enriched. Dot plots were created to visualize significant activated and suppressed pathways using the dotplot function from the R package enrichplot v1.14.2 (33).

<u>NK92 Dusp1 overexpressing cell line creation</u>

The Sleeping Beauty transposon system expressed Dusp1 in NK92 cells purchased from ATCC (#CRL-2407). An empty vector control cell line was created as a corresponding control.

Sleeping Beauty vectors construction: The backbone transposon plasmid pT2/HB was a gift from Perry Hackett (University of Minnesota, Minneapolis, MN 55455, USA., Addgene plasmid # 26557). The transposase plasmid pCMV(CAT)T7-SB100 was a gift from Zsuzsanna Izsvak (Max-Delbrück-Centrum für Molekulare Medizin (MDC) Robert-Rössle-Straße 10, 13125 Berlin, Germany, Addgene plasmid # 34879). The *DUSP1* gene ORF (Ensembl, ENSG00000120129) was synthesized from GeneArt (Thermo Fisher) and was cloned into pT2/HB vector through In-Fusion HD Cloning Plus kit (Takara Bio) following manufacturers' instructions.

Electroporation of NK-92 cells: NK-92 cells were harvested, counted, and resuspended in 100 uL Cell Line Nucleofector Solution R (Lonza) at the concentration of 1×10^8 cells/mL. pT2/HB transposon plasmids encoding DUSP1 or truncated CD34 (16 ug) and pCMV(CAT)T7-SB100 transposase plasmids (4ug) were added for each electroporation based on optimization experiments. The electroporation was performed on Nucleofector 2b (Lonza) using the program A-024. Cells were returned to the incubator for 48 hours and resuspended at the desired concentration with fresh complete medium. On day 7 post electroporation, CD34+ cells were sorted using anti-human CD34 magnetic beads (Miltenyi). The percentage of CD34+ cells before and after sorting was measured using another anti-human CD34 mAb (Miltenyi, clone AC136) binding a different epitope than the one recognized by anti-human CD34 magnetic beads. The sorted cells were expanded and used for phenotyping and functional assays.

Supplementary References:

1. Turner RJ, et al. The Epidemiology of Social Stress. *American Sociological Review.* 1995;60(1):104-25.
2. Eaton WW, et al. *In M E Maruish (Ed), The use of psychological testing for treatment planning and outcomes assessment: Instruments for adults*. Lawrence Erlbaum Associates Publishers; 2004:363-77.
3. Baumer Y, et al. Immune cell phenotyping in low blood volumes for assessment of cardiovascular disease risk, development, and progression: a pilot study. *J Transl Med.* 2020;18(1):29.
4. Aguilar Díaz de León JS, et al. Oxidized-Desialylated Low-Density Lipoprotein Inhibits the Antitumor Functions of Lymphokine Activated Killer Cells. *J Cancer.* 2021;12(16):4993-5004.
5. Malorni W, et al. Oxidized Low-Density Lipoproteins Affect Natural Killer Cell Activity by Impairing Cytoskeleton Function and Altering the Cytokine Network. *Experimental Cell Research.* 1997;236(2):436-45.
6. De Sanctis JB, et al. Expression and function of low-density lipoprotein receptors in CD3-CD16+CD56+ cells: effect of interleukin 2. *Cell Immunol.* 1996;167(1):18-29.
7. Baumer Y, et al. Hyperlipidaemia and IFNgamma/TNFalpha Synergism are associated with cholesterol crystal formation in Endothelial cells partly through modulation of Lysosomal pH and Cholesterol homeostasis. *EBioMedicine.* 2020;59:102876.
8. Baumer Y, et al. Hyperlipidemia-induced cholesterol crystal production by endothelial cells promotes atherogenesis. *Nat Commun.* 2017;8(1):1129.
9. Baumer Y, et al. Chronic skin inflammation accelerates macrophage cholesterol crystal formation and atherosclerosis. *JCI Insight.* 2018;3(1).
10. Gutierrez-Huerta CA, et al. LDL associates with pro-inflammatory monocyte subset differentiation and increases in chemokine receptor profile expression in African Americans. *Int J Cardiol.* 2022;358:88-93.
11. Virani SS, et al. 2023 AHA/ACC/ACCP/ASPC/NLA/PCNA Guideline for the Management of Patients With Chronic Coronary Disease: A Report of the American Heart Association/American College of Cardiology Joint Committee on Clinical Practice Guidelines. *Circulation.* 2023;148(9):e9-e119.
12. Baumer Y, et al. CD98 regulates vascular smooth muscle cell proliferation in atherosclerosis. *Atherosclerosis.* 2017;256:105-14.
13. Baumer Y, et al. Neighborhood socioeconomic deprivation and individual-level socioeconomic status are associated with dopamine-mediated changes to monocyte subset CCR2 expression via a cAMP-dependent pathway. *Brain Behav Immun Health.* 2023;30:100640.
14. Kuhn S, and Tandon M. Zenodo; 2024.
15. Andrews S. FastQC: A Quality Control Tool for High Throughput Sequence Data [Online]. Available online at: http://www.bioinformatics.babraham.ac.uk/projects/fastqc/. 2010.
16. Daley T, and Smith AD. Predicting the molecular complexity of sequencing libraries. *Nat Methods.* 2013;10(4):325-7.
17. Tools P. Broad Institute, GitHub repository. http://broadinstitute.github.io/picard/.
18. Wingett SW, and Andrews S. FastQ Screen: A tool for multi-genome mapping and quality control. *F1000Res.* 2018;7:1338.

19. Wood DE, et al. Improved metagenomic analysis with Kraken 2. *Genome Biol.* 2019;20(1):257.
20. García-Alcalde F, et al. Qualimap: evaluating next-generation sequencing alignment data. *Bioinformatics.* 2012;28(20):2678-9.
21. Wang L, et al. RSeQC: quality control of RNA-seq experiments. *Bioinformatics.* 2012;28(16):2184-5.
22. Martin M. Cutadapt removes adapter sequences from high-throughput sequencing reads. *2011.* 2011;17(1):3.
23. Frankish A, et al. GENCODE: reference annotation for the human and mouse genomes in 2023. *Nucleic Acids Research.* 2022;51(D1):D942-D9.
24. Dobin A, et al. STAR: ultrafast universal RNA-seq aligner. *Bioinformatics.* 2012;29(1):15-21.
25. Li B, and Dewey CN. RSEM: accurate transcript quantification from RNA-Seq data with or without a reference genome. *BMC Bioinformatics.* 2011;12(1):323.
26. Robinson MD, et al. edgeR: a Bioconductor package for differential expression analysis of digital gene expression data. *Bioinformatics.* 2010;26(1):139-40.
27. Kolde R. pheatmap: Pretty Heatmaps. R package version 1.0.12. https://CRAN.R-project.org/package=pheatmap. 2019.
28. Kassambara A. ggpubr: 'ggplot2' Based Publication Ready Plots. R package version 0.4.0. https://CRAN.R-project.org/package=ggpubr. 2020.
29. Law CW, et al. voom: precision weights unlock linear model analysis tools for RNA-seq read counts. *Genome Biology.* 2014;15(2):R29.
30. Blighe K, et al. EnhancedVolcano: Publication-ready volcano plots with enhanced colouring and labeling. R package version 1.12.0. https://github.com/kevinblighe/EnhancedVolcano. 2021.
31. Wu T, et al. clusterProfiler 4.0: A universal enrichment tool for interpreting omics data. *Innovation (Camb).* 2021;2(3):100141.
32. Dolgalev I. msigdbr: MSigDB Gene Sets for Multiple Organisms in a Tidy Data Format. R package version 7.5.1. https://CRAN.R-project.org/package=msigdbr. 2022.
33. Yu G. enrichplot: Visualization of Functional Enrichment Result. R package version 1.14.2. https://yulab-smu.top/biomedical-knowledge-mining-book/. 2022.
