# Supplementary material for: Obesity modulates NK cell activity via LDL and DUSP1 signaling for populations with adverse social determinants
Source: JCI Insight. 2024 Dec 24;10(2):e180606. doi: 10.1172/jci.insight.180606 (PMC11790026; doi:10.1172/jci.insight.180606)

Full Unedited Blot for Figure 3C – NFkB Western Blot

1x blot with 3 samples and ladder; both in duplicate; blot cut vertically in half; left probed for NFkB; right probed for P-NFkB

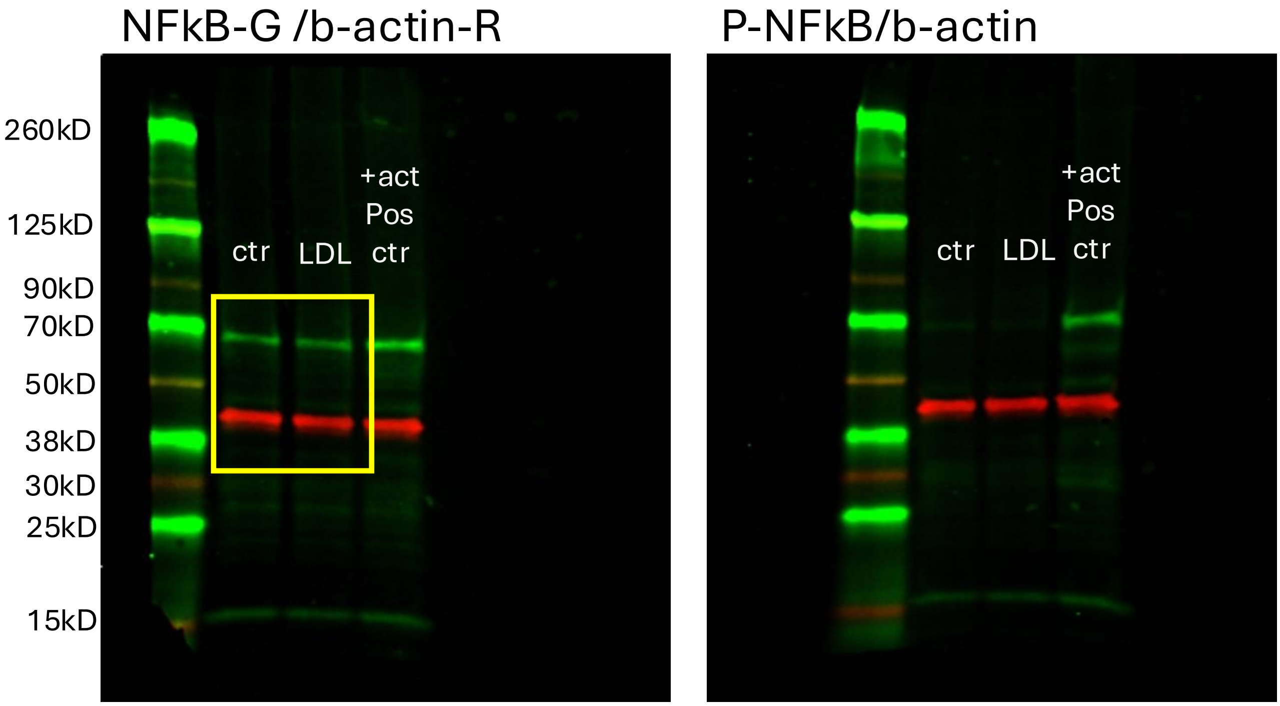

Full Unedited Blot for Figure 3D – NFkB Western Blot

1x blot with 8 samples and ladder; cut horizontally to allow for detection of various proteins

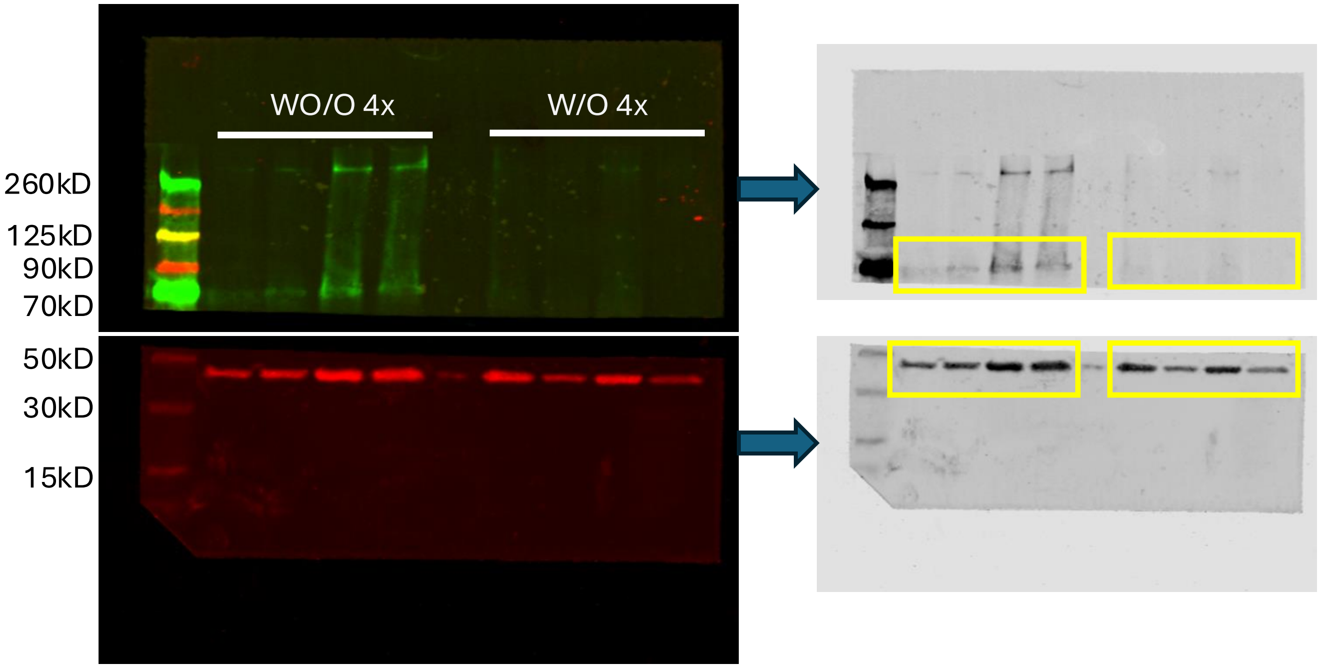

Full Unedited Blot for Figure 3E – P62

1x blot with 8 samples and ladder; Top used for p62-Green/b-actin-Red (bottom was used for detection of a different protein

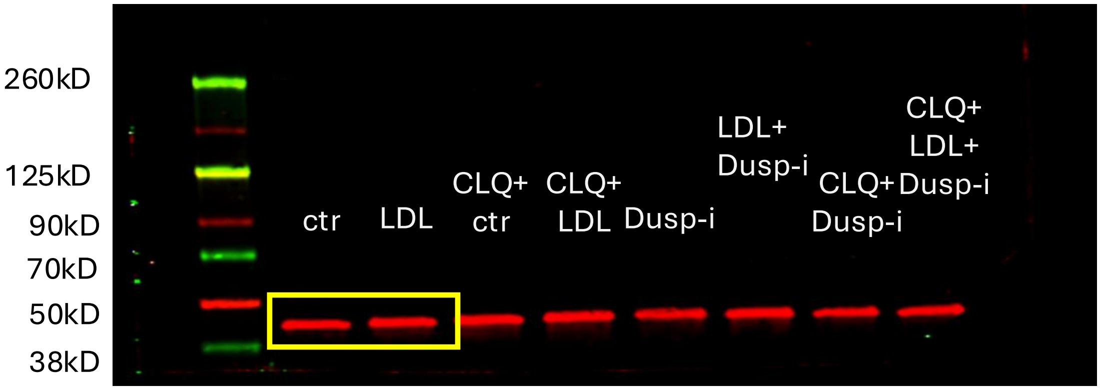

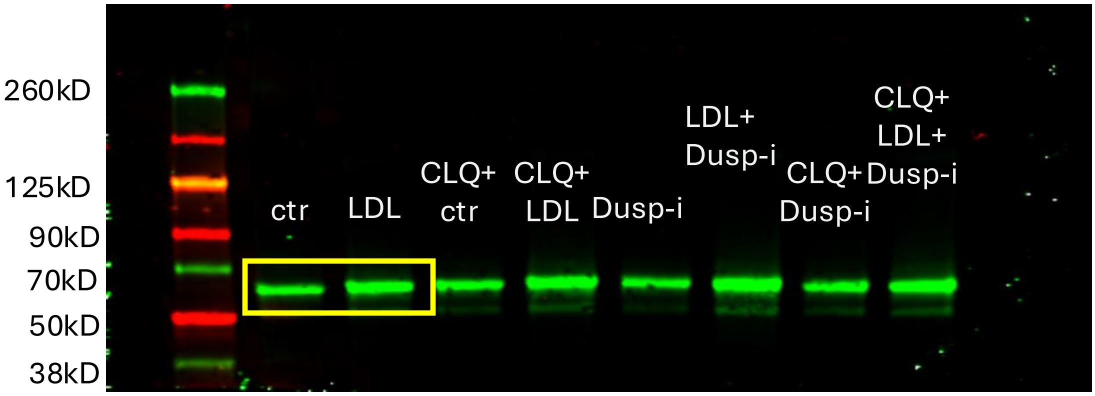

Full Unedited Blot for Figure 3F – LC3 – autophagic flux

1x blot with 8 samples and ladder; Top used for b-actin; bottom was used for detection of a LC3

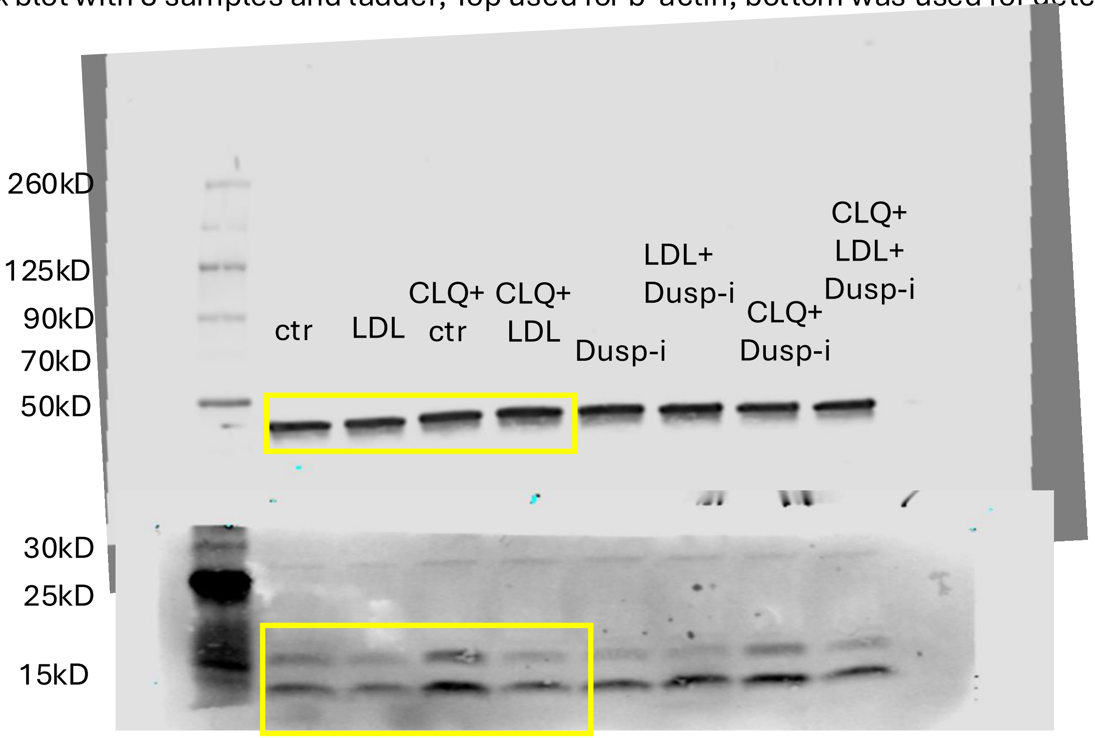

Full Unedited Blot for Figure 6E – LC3 – autophagic flux

1x blot with 6 samples and ladder; Top used for b-actin-Red and TFEB-Green; bottom was used for detection of a LC3-Green

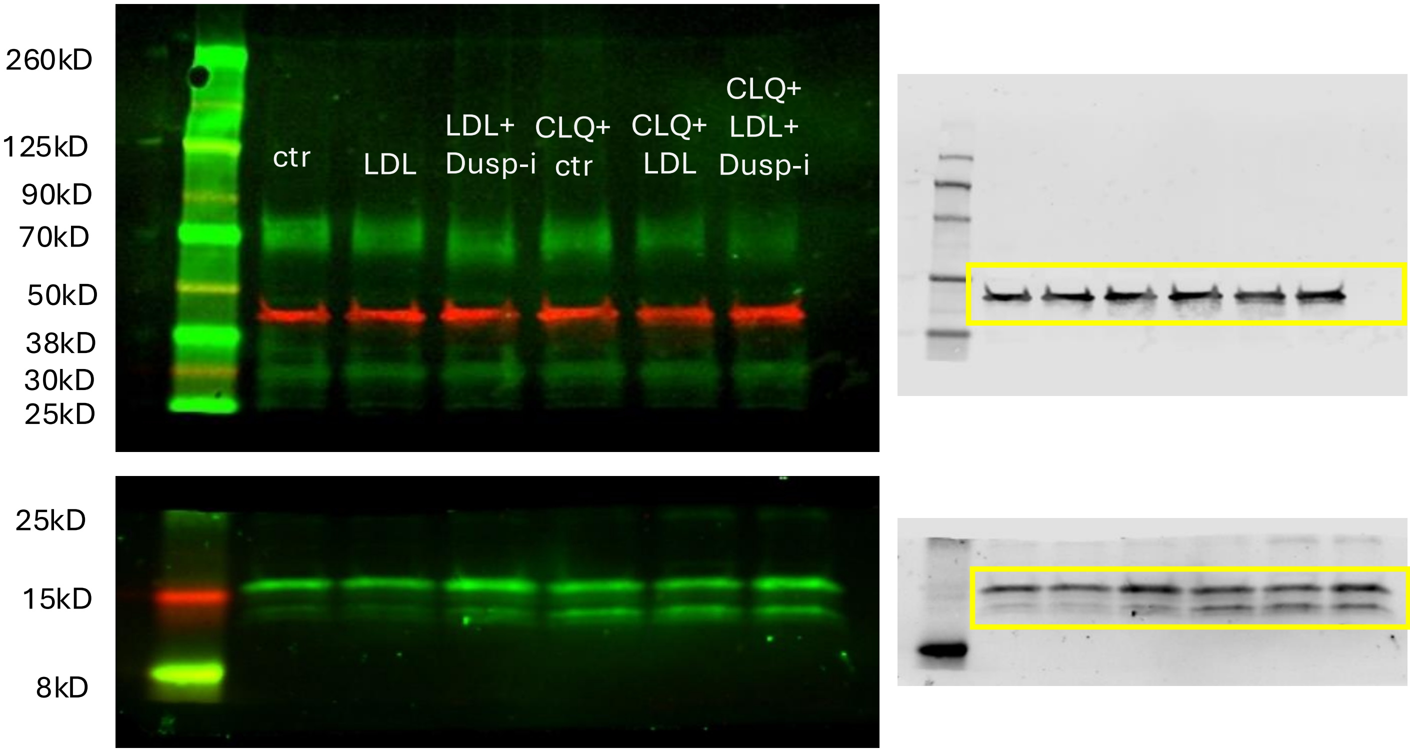

Full Unedited Blot for Figure 6H – TFEB blot

1x blot with 9samples and ladder (3 sets of 3 treatments each; Top above 125kDa used for different protein; bottom <125kDa used for b-actin-Red and TFEB-Green; set 2 is shown in the graph (middle)

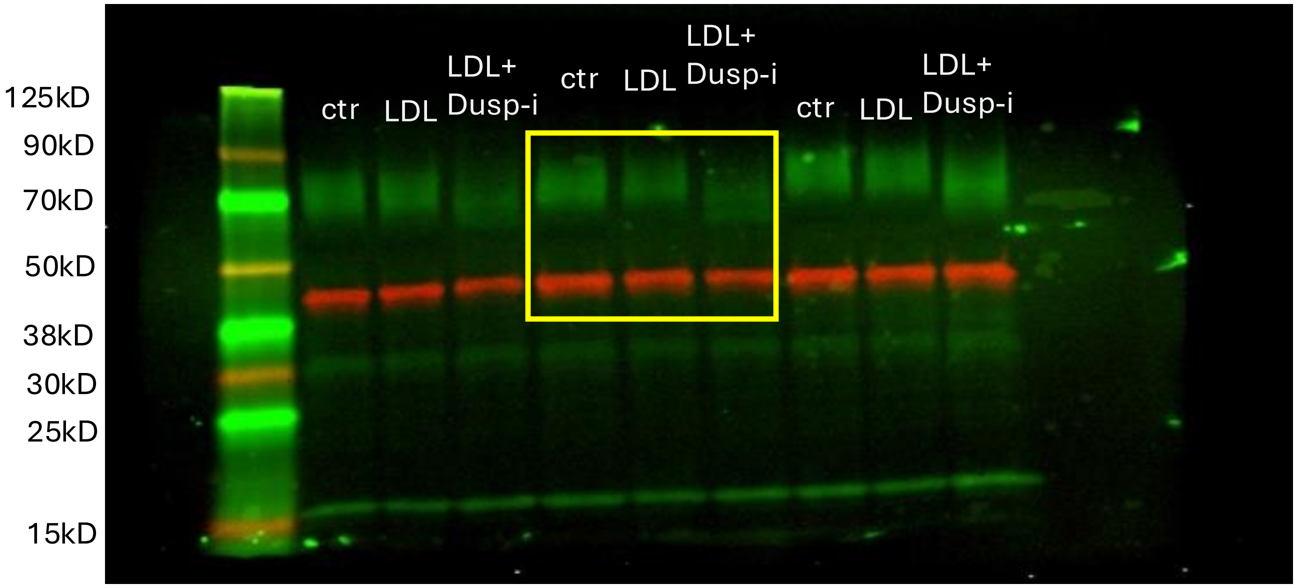

Full Unedited Blot for Figure 6K – TFEB and P-TFEB

2x blots with the same samples in the same order; 4 lean individuals and 5 individuals with obesity
blot 1: TFEB-Green and b-actin-Red
Blot 2:P-TFEB-Green and b-actin-Red
Both blots cut at 125kDa (>125kDa used for different protein

Blot1

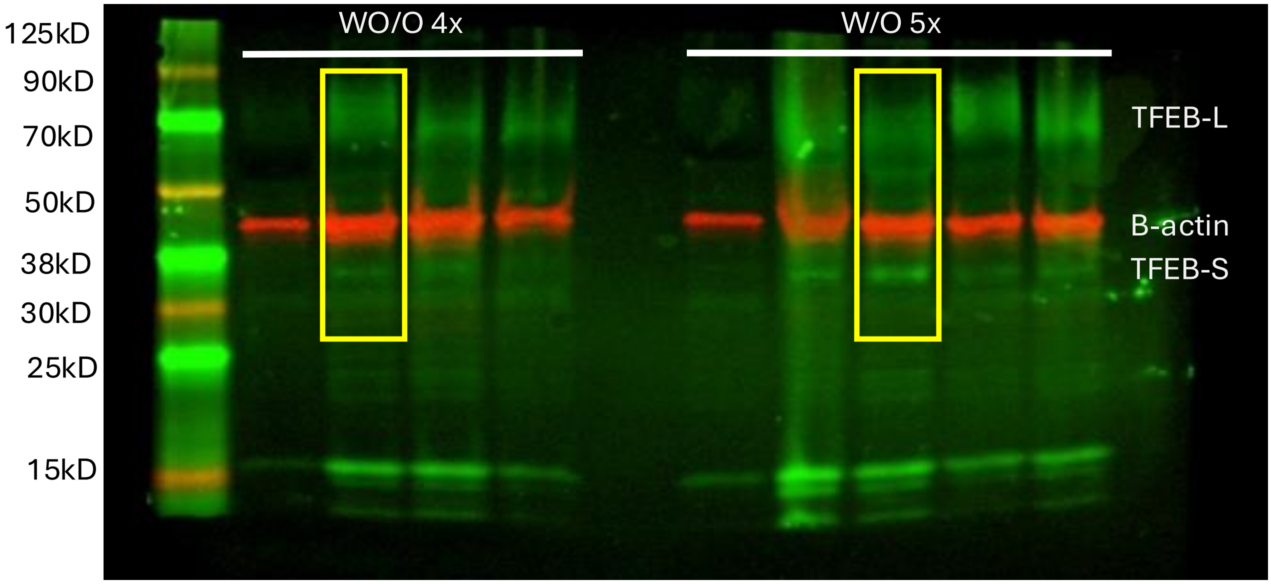

Blot2 – not pictured in Figure

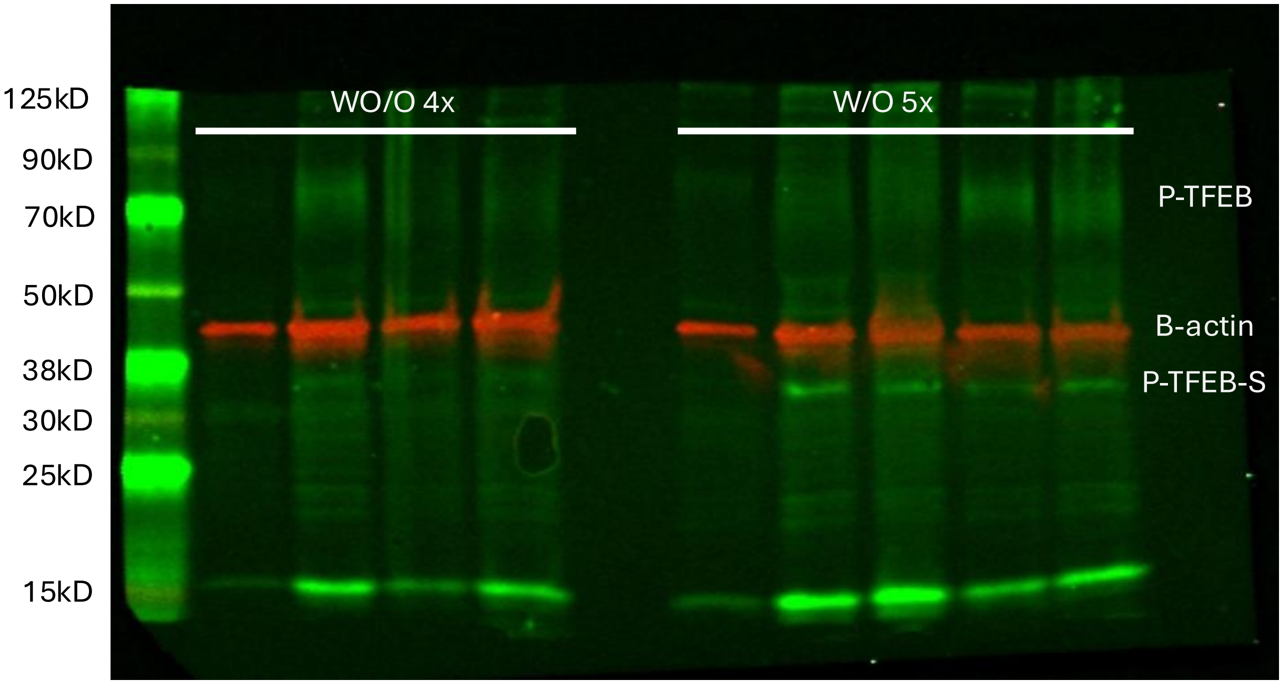

Supplement: Unedited blot and gel images [file jciinsight-10-180606-s226.pdf]
